# Wireless Power and Data Transfer over Inductive Links for Biomedical Applications: Recent Advances, Challenges, and Limitations

**DOI:** 10.3390/mi17090998

**Published:** 2026-08-24

**Authors:** Naqeeb Ullah, Adel Barakat, Haruichi Kanaya

**Affiliations:** 1 Graduate School of Information Science and Electrical Engineering, Kyushu University, Fukuoka 819-0396, Japan; abarakat@esyncssb.co.jp (A.B.); kanaya@ed.kyushu-u.ac.jp (H.K.); 2 Electronic Engineering Department, Faculty of Information and Communication Technology, Balochistan University of Information Technology, Engineering and Management Sciences (BUITEMS), Quetta 87300, Balochistan, Pakistan; 3 eSync SSB, Inc., Fukuoka 814-0001, Japan

**Keywords:** biomedical, downlink, inductive link, IMDs, uplink, power transfer efficiency, wireless power transfer

## Abstract

Wireless power transfer (WPT) has emerged as a promising technology for modern implantable medical devices (IMDs), providing a reliable alternative to traditional batteries and reducing the need for repeat surgical procedures. WPT-based IMDs are extensively employed in biomedical applications such as deep brain stimulators, endoscopic capsules, pacemakers, and cardiac monitoring systems, which are being commercialized. As the IMD market is expected to grow with the aging population, extensive research efforts are proceeding to address key design and implementation challenges. A key challenge lies in simultaneously achieving high power-transfer efficiency (PTE), high data rates, low power consumption, and low circuit complexity. This review paper thoroughly examines recent developments, design trends, and current research problems in simultaneous power and bidirectional data transmission over a single inductive link, as well as the associated modulation and demodulation techniques. We reviewed various uplink and downlink modulation techniques, focusing on key characteristics such as data rate, power consumption, circuit complexity, and health and safety considerations. Finally, future research directions toward efficient, compact, and reliable simultaneous wireless power and data transfer (SWPDT) for the next generation of IMDs are discussed. This review paper will hopefully guide ongoing research into more efficient, scalable, and safe SWPDT systems.

## 1. Introduction

**Figure 1:**
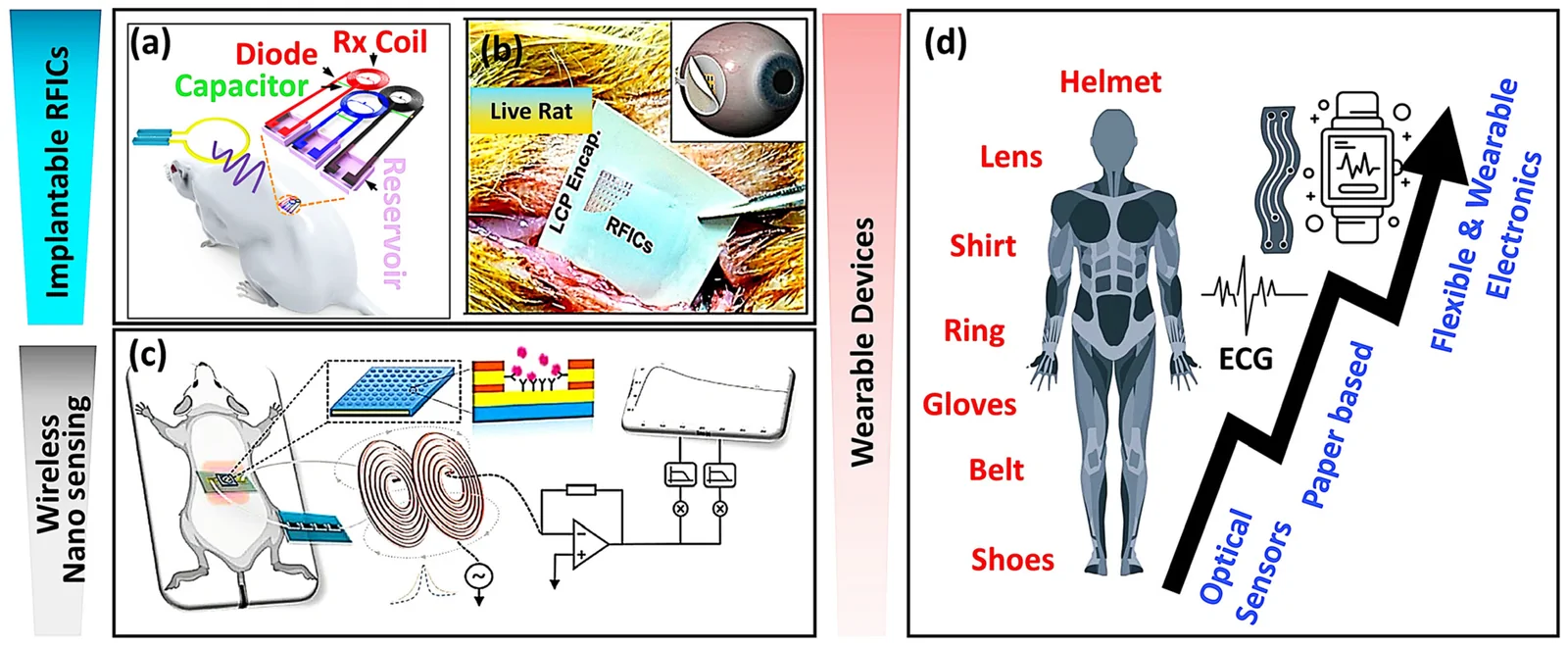
(**a**) Drug delivery systems enable high-efficiency delivery of drugs to specific sites and precise real-time control of release and dosing. Reproduced from Ref. [9]. (**b**) Flexible RF-IC devices with LCP encapsulation embedded between skin and underlying tissue of a rat. Reprinted/adapted with permission from Ref. [18]. (**c**) A wireless biosensor system has been developed for the real-time measurement of protein biomarkers in vivo using resonant inductive coupling. Reprinted/adapted with permission from Ref. [10] under the Creative Commons Attribution (CC BY 4.0) License. Copyright © 2025 The Authors. Published by the American Chemical Society. (**d**) Smart wearable devices used for real-time continuous monitoring of physiological signals. Reprinted/adapted with permission from Ref. [11].

In the modern healthcare system, many advanced medical devices have been developed for diagnosis and treatment, including smart drug delivery systems, biosensors, and wearable and portable devices [1,2,3,4,5,6,7,8,9,10,11]. Among them, implantable medical devices (IMDs), including cochlear implants, cardiac pacemakers, intraocular pressure monitors, and drug delivery devices, are implanted in the body to enable continuous monitoring and targeted intervention [12,13,14,15,16,17,18], as illustrated by typical examples in Figure 1. To enable long-term operation inside the body, recent research has focused on improving the power efficiency, flexibility, and autonomy of such systems. As shown in Figure 1a, a wireless bioresorbable drug delivery system with multiple independently addressable reservoirs enables precise, on-demand drug release via RF harvesting at different resonant frequencies. Flexible bio-integrated electronics based on ultrathin silicon RF-ICs encapsulated with liquid crystal polymer (LCP) overcome the limitations of rigid implants, providing better mechanical compliance, biocompatibility, and reliable in vivo wireless communication. Figure 1c shows a wireless biosensing system based on resonant inductive coupling for real-time, label-free monitoring of protein biomarkers, eliminating the need for wired connections and enabling continuous operation. This technique enables remote impedance measurements of a nano well array without wired connections, enabling continuous, non-invasive monitoring. Figure 1d illustrates the role of smart wearable biosensors in continuous physiological monitoring under ambulatory conditions. All these works highlight the integration of miniaturization, system flexibility, and wireless power and data transfer techniques, which are critical to enabling low power, reliability, and continuous operation in advanced IMD systems. The main barrier to long-term IMD performance is delivering reliable power and data within the body. Battery-based solutions are limited by short lifespan and the need for surgical replacement, which can lead to complications such as infection and delayed patient recovery. Wireless power transfer (WPT) has been used as an alternative to batteries to deliver energy safely and effectively to implanted devices within the body without a physical connection. For real-time monitoring and control operations, such as precise therapy, IMDs require sufficient energy and data transfer capabilities.

**Figure 2:**
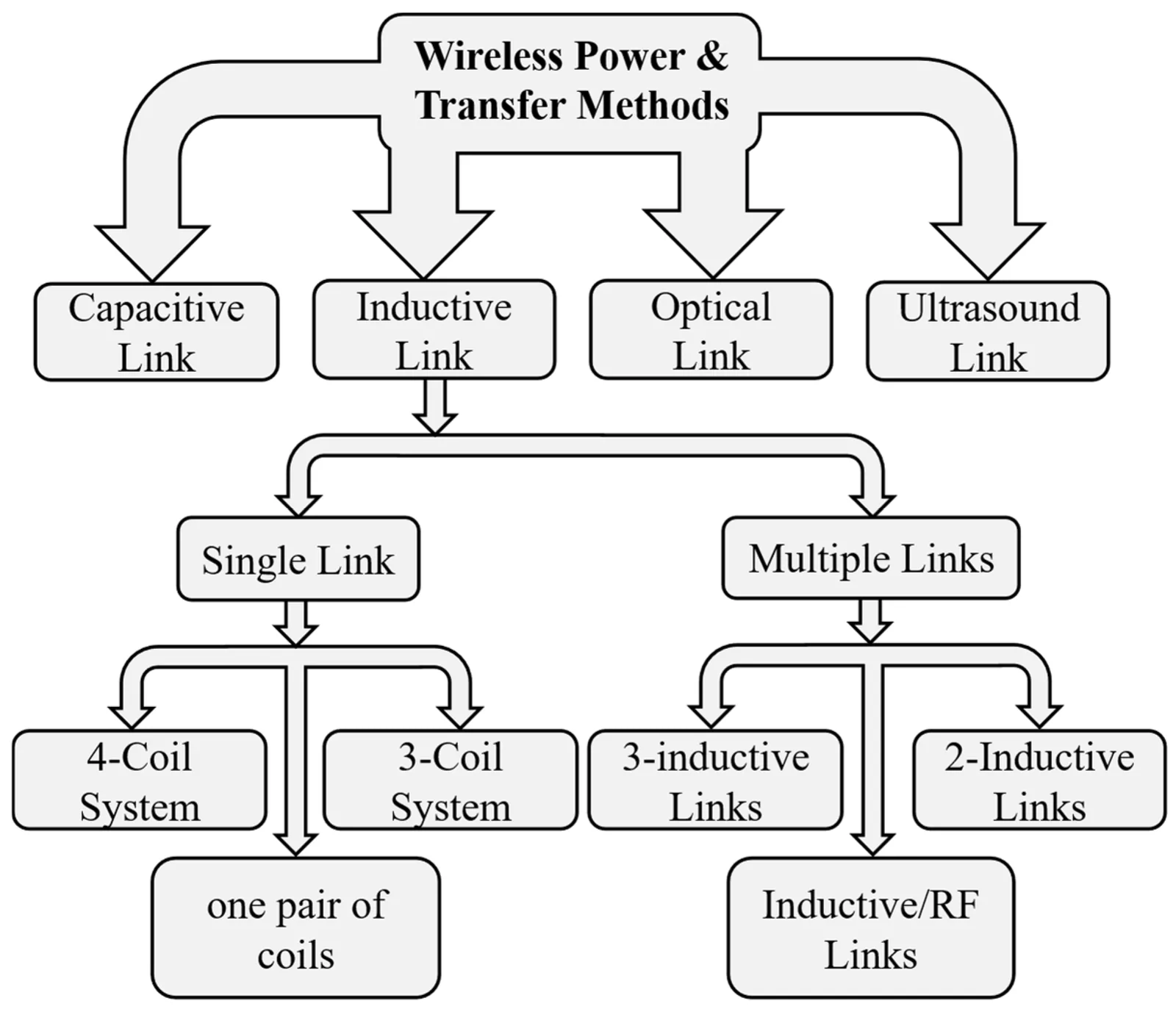
Summary of various wireless power transfer methods.

Several WPT techniques have been utilized for wireless power transfer, as illustrated in Figure 2. Among them are inductive coupling, ultrasonic, optical, and capacitive link techniques [12,13,14,15,16,17,18,19,20,21,22,23]. These are well-known techniques. Each has its own advantages and limitations depending on its application. Inductive and ultrasound have higher efficiency and penetration, while optical offers high data rates but low penetration, and capacitive works only over short distances. Inductive links are the best choice for biomedical applications due to safe interaction with tissue and reliable operation. The inductive WPT system can be categorized into single inductive links and multiple inductive links. In the single inductive link category, the 3-coil and 4-coil systems refer to architectures with additional resonator coils to improve PTE while maintaining a single inductive link between the transmitter and receiver. The multiple inductive link systems provide 3-coil systems and 4-coil systems with one and two additional resonator coils, respectively. In biomedical implants, the WPT system has several key factors, such as high-power transfer efficiency (PTE) for efficient power delivery, maintaining sufficient data rates for communication, minimizing power consumption to avoid overheating, and extending operating time. Moreover, the circuit design should be small enough for integration with other systems. Inductive links provide the best trade-off among these requirements.

In this paper, we reviewed recent advances in wireless power and data transfer using inductive links for biomedical implants. Numerous studies were shortlisted through major scientific databases, mainly IEEE Xplore, Scopus, and Web of Science. Relevant studies were identified by keywords such as implantable devices, inductive links, inductive coupling, wireless power transfer, forward data transfer, back data transfer, ASK, FSK, CWM, LSK, OOK, and modulation techniques from 2000 to 2026. The literature is analyzed with respect to communication architecture, circuit implementation, power consumption, data rate, and overall system key performance.

Several review papers have been published focusing on major advances in inductive link-based WPDT architectures for biomedical implants. Among them, the work of Trigui et al. [23] primarily focused on single-coil inductive-link designs, while Karimi et al. [24] reviewed different modulation and telemetry methods. On the other hand, in [25], the authors focus on theoretical considerations and discussion of link optimization approaches. However, a broad discussion on recently proposed methods such as Carrier Wave Modulation (CWM) and Quadrature Carrier Wave Modulation (QCWM) and on–off keying was not highlighted. Moreover, significant advances have been made in the field over the last few years, particularly in terms of communication reliability and power transfer efficiency. This review aims to bridge these gaps by offering in-depth coverage of recent advances in single-coil and multiple-coil inductive link architectures, reviewing emerging communication techniques, and describing the transition of inductive link technologies from research prototypes to clinically relevant, commercialized systems.

The remainder of the paper is structured as follows. Section 2 describes the basic principles of wireless power and data transfer systems, including single and multiple inductive-link structures for biomedical implant applications. Section 3 reviews the downlink data communication techniques employed over inductive links, including ASK, OOK, CW, QCWM, FSK, PSK, PDM, and PHM schemes, and provides a comprehensive evaluation of key performance metrics. In Section 4, uplink data communication based on a single inductive link is proposed, and in Section 5, full-duplex communication systems, which allow simultaneous bidirectional data transfer, are introduced. The validation status of the reviewed systems is summarized in Section 6. Section 7 presents the safety guidelines and regulatory considerations for WPDT systems. Finally, Section 8 presents the key challenges, emerging trends, and future directions for research in WPDT technologies for implantable biomedical devices.

## 2. Wireless Power and Data Transfer via Inductive Links in Biomedical Implants

Inductive coil-based WPT is one of the most widely adopted techniques for delivering power to implantable biomedical devices due to its safety and reliable performance in near-field operation. The time-varying magnetic field generated by the external primary coil induces a voltage in the implanted secondary coil, enabling direct power transfer. The key factors that determine the power transfer efficiency (PTE) are the coupling coefficient (k) and quality factor (Q), as given in Equations (1) and (2):

(1) $$k=\frac{M}{\sqrt{L_{1}L_{2}}}$$

(2) $$Q=\frac{2\pi fL}{R_{\mathrm{eff}}}$$

M denotes the mutual inductance between two inductive coils, and the self-inductances of the primary and secondary coils are represented by L_1_ and L_2_, respectively. In Equation (2), L and $R_{\mathrm{eff}}$ are self-inductance and resistance, while $Q_{1}$ and $Q_{2}$ are quality factors for two coils, and $Q_{2L}=Q_{2}Q_{L}/Q_{2}{+\;Q}_{L}$. The PTE is highly dependent on both the coil size and the distance between the primary and secondary coils. Adopted from [26], Equation (3) is derived from a two-coil series-parallel (S/P) resonant compensated inductive link and assumes the optimal load matching condition that maximizes the PTE is expressed in Equation (3):

(3) $$\eta =\frac{k^{2}Q_{1}Q_{2L}}{{\left(1+\sqrt{k^{2}Q_{1}Q_{2L}}\right)}^{2}}$$

It also depends on precise alignment between the two coils. The inductive WPT link can be categorized into multiple links and a single inductive link. In a multiple-link coil system, either a dedicated inductive channel for transferring power and data or a combination of inductive links and RF is used. In a single inductive link, a single link carries both power and data. This section presents a multi-inductive and single-inductive link system for power and data transfer.

### 2.1. Multiple Inductive Links

To improve data rate, multiple inductive links are preferred, as they use separate coil pairs for power and data. This method allows the uplink and downlink to use a different pair of coils. This method ensures stable cases when space and power economy are critical; designers must balance the benefits of flexible communication against the challenges posed by rising hardware complexity and physical constraints. However, this approach increases system complexity, size, and cost, as it requires additional coils and proper alignment.

**Figure 3:**
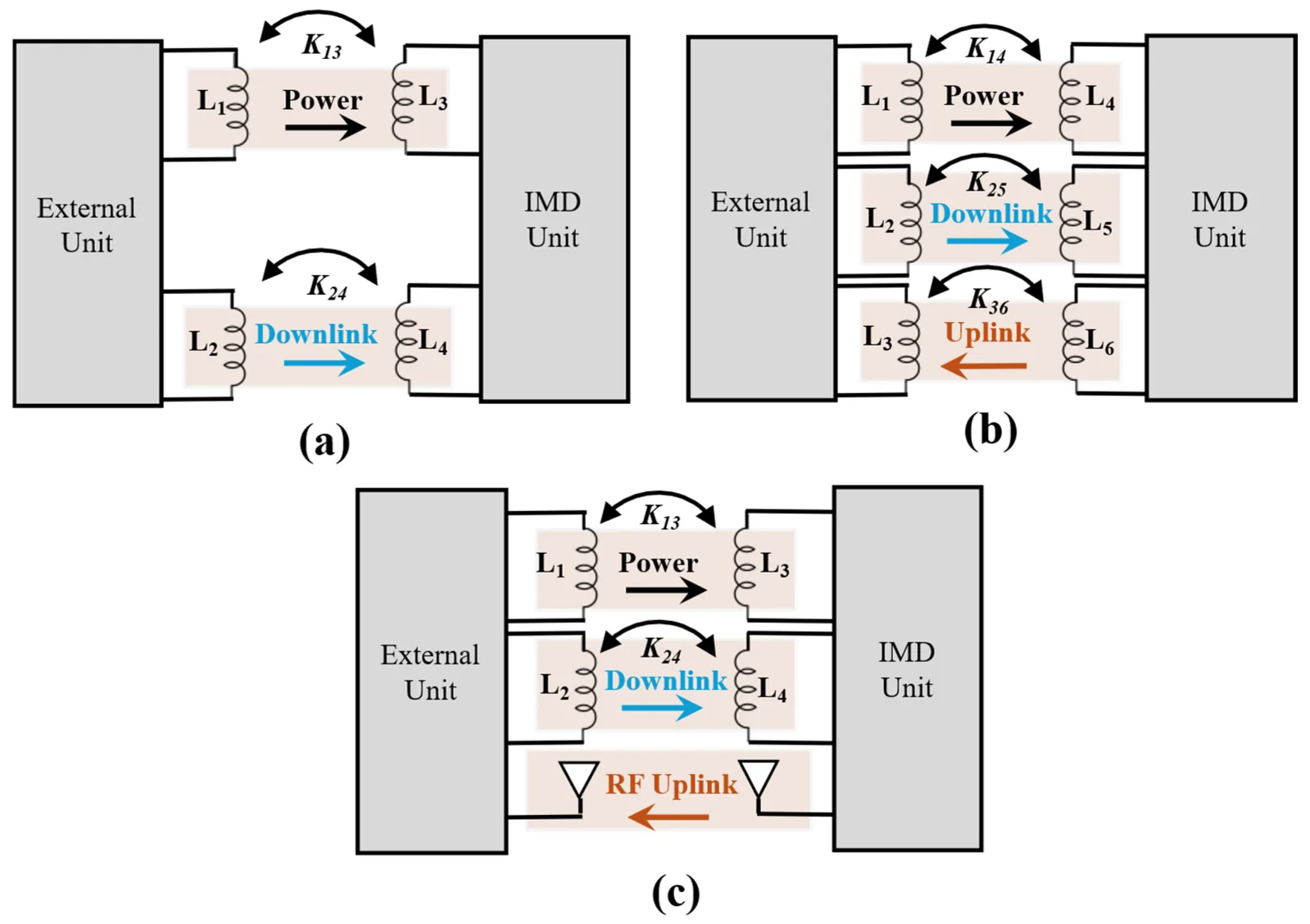
Multiple inductive link-based wireless power and data transfer: (**a**) Two inductive links, (**b**) three inductive links, (**c**) dual coupled inductive links with uplink RF link.

Figure 3 shows multiple inductive links that enable wireless power and data transfer. Compared with a single-link architecture, multiple-inductive-link systems achieve higher data rates by using separate coils to transfer power and data. The data channels are split into downlinks for sending commands to control stimulation and uplinks for sending telemetry of physiological parameters or IMD status to the outside unit. The system architectures vary in their use of distinct links for power and unidirectional or bidirectional data transmission, as shown in Figure 3a,b [12,17,18,19]. Some systems also use RF antennas for uplink or back telemetry, as illustrated in Figure 3c, which offers an extended communication range but consumes high power. Multi-link systems improve bandwidth and reliability but are more complex to design. This makes it harder to manage electromagnetic coupling to reduce coil crosstalk.

**Figure 4:**
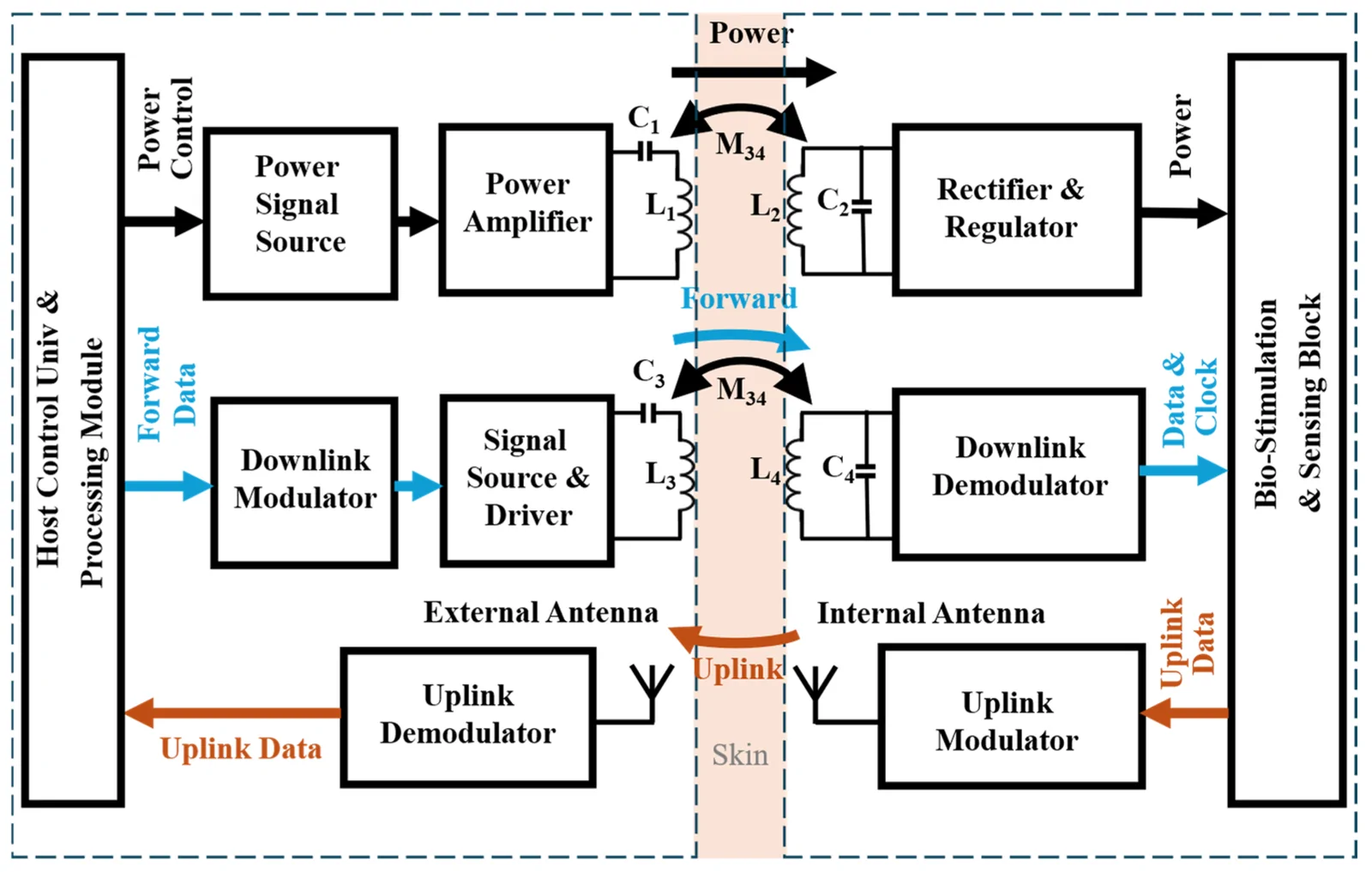
Block diagram of wireless power, uplink, and downlink data transfer.

Figure 4 presents a block diagram of a combined inductive link and radio frequency system designed for simultaneous wireless data and power transfer. Two inductively coupled coil systems facilitate power delivery and forward data, while uplink data transmission is achieved via a radio-frequency antenna. The topology consists of the external unit that includes a power source, controller, power amplifier, and modulator and demodulator circuits. The power supply energizes the power amplifier, which energizes the primary coil to generate an alternating magnetic field for inductive powering. The controller manages all operations, including power transmission, downlink command modulation, and uplink data receiving. The modulator puts data or control instructions on the transmitted signal for the IMD, whereas the downlink demodulator extracts physiological or system-related feedback from the implant. On the receiver side, the incoming modulated signal is first passed through a power conversion chain comprising a rectifier and a voltage regulator. The rectifier converts the received AC power into a DC voltage, which is fed to the voltage regulator to stabilize the supply and meet the requirements of internal circuits. To transfer physiological data, such as neural signals, back to the external unit, an uplink modulator is used. Either a dedicated RF antenna or an inductive link is used. These signals are decoded by the uplink demodulator at the transfer side. This architecture enables reliable, miniaturized, and bidirectional data transfer for biomedical applications, including power transmission, downlink command modulation, and uplink data receiving. The modulator puts data or control instructions on the transmitted signal for the IMD, whereas the downlink demodulator extracts physiological or system-related feedback from the implant.

### 2.2. Single Inductive Link

**Figure 5:**
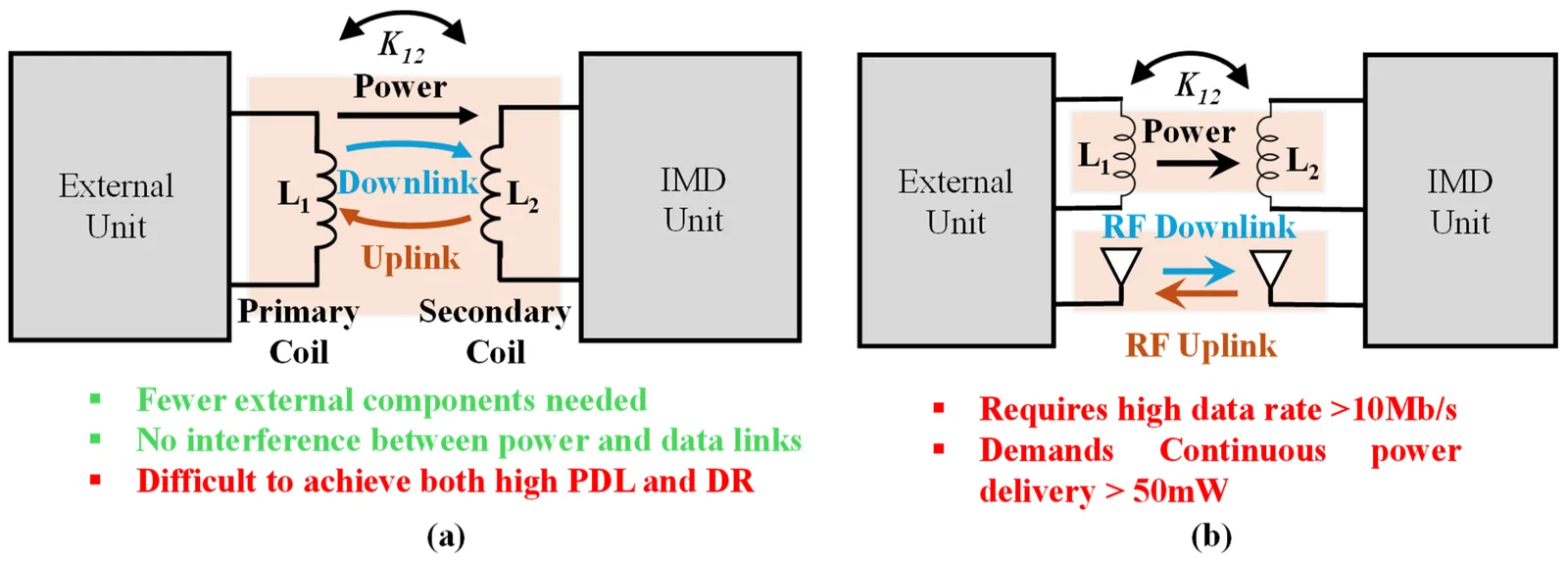
Simultaneous wireless power and data transfer: over inductive coils: (**a**) single inductive link and RF link approach, (**b**) single inductive link for both power and bidirectional data transfer.

The simplest way to achieve SWPDT over an inductive link is to employ a single coupled-coil system, as illustrated in Figure 5. This method reduces system complexity, size, and cost while limiting human exposure to EM fields, making it safer and more reliable [20,21,22]. A single inductive link utilizes few components to enable simultaneous power and data transfer, with no interference between them, as shown in Figure 5a. In contrast, a single inductive coil with an RF antenna demands high bandwidth (BW), as illustrated in Figure 5b. Moreover, transmitting power and data over a single inductive link faces challenges due to inherent trade-offs between coil quality factors (Q), bandwidth (BW), and carrier frequency (f). A high Q increases power-transfer efficiency by reducing resistive losses but limits bandwidth, thereby reducing data rate and tolerance to frequency variations. Conversely, increasing bandwidth to support higher data rates demands lowering Q, which reduces efficiency. The operating frequency also affects coil size and losses. Higher frequencies enable smaller coils to raise skin and proximity effects, leading to higher resistance and reduced Q.

To improve a single inductive link, a balancing strategy is required to optimize efficiency, bandwidth, and coil size according to application demands. For example, biomedical implants require a compact, reliable inductive system, while consumer electronics require a higher data rate. The relationships between BW, Q, and $f_{c}$ are shown in Equation (4) [23].

(4) $$BW=f_{c}/Q$$

Additionally, a single-pair coil system also suffers from weak coupling and sensitivity to misalignment, resulting in reduced efficiency and mismatched power distribution [24]. To address these challenges, high-Q resonators are introduced between the primary and secondary coils in a multi-coil configuration. Three-coil and four-coil systems significantly improve efficiency compared to single-link systems [25,26,27]. For instance, the 3-coil system adds an external transmitter, a high-Q resonator, and an implanted receiver to enhance power transfer in brain implants, with the transmitter and resonator positioned externally. Similarly, the 4-coil systems further improve efficiency by adding additional inductive coils [17,28]. Despite these improvements, multi-coil designs increase system size, which remains a critical limitation for implantable applications, where coil dimensions can range from 1.2 mm to 32 mm.

## 3. Data Telemetry and Modulation Techniques over a Single Inductive Coil System

**Figure 6:**
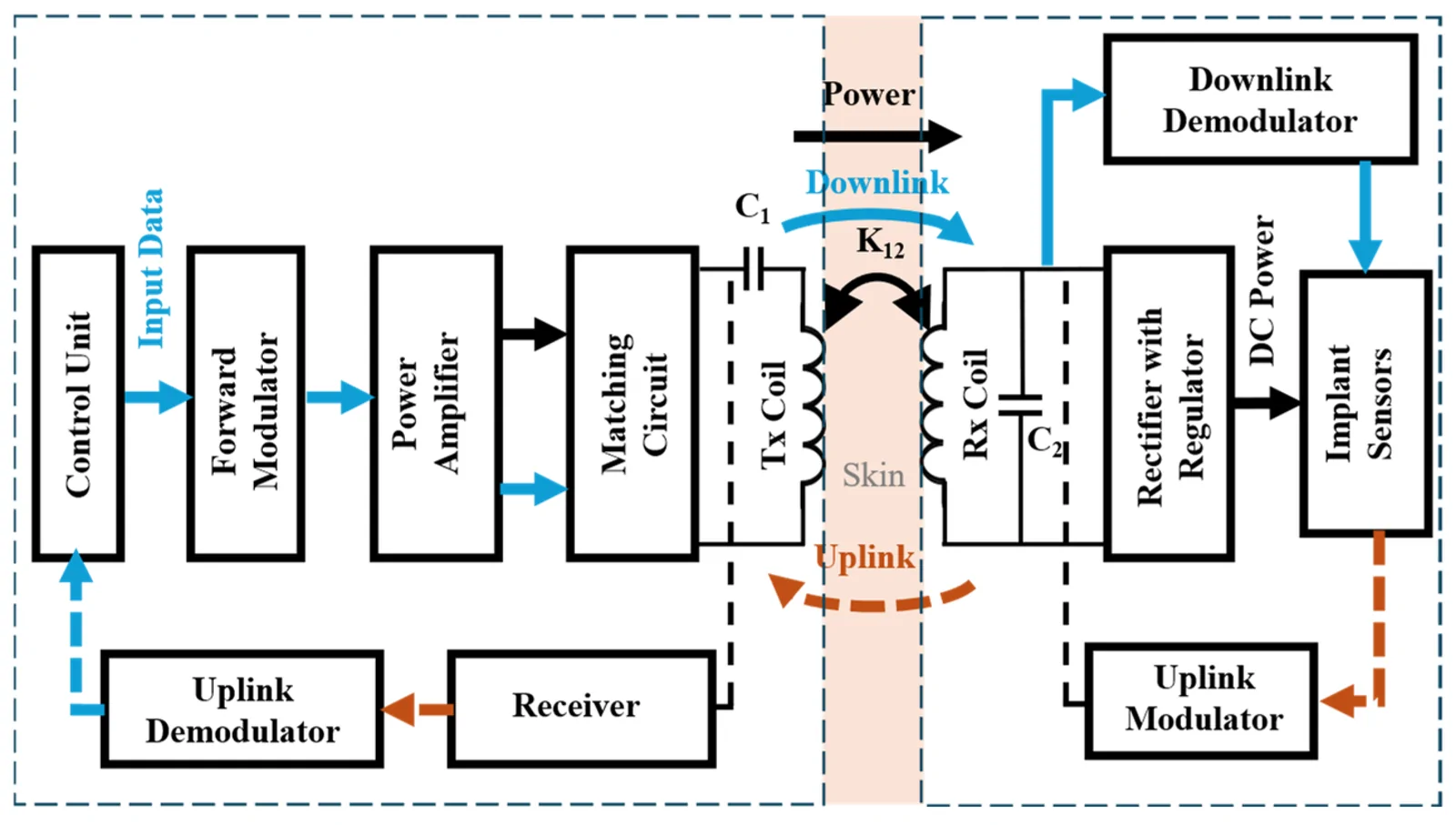
Data and power transfer system using a single inductively coupled coil link.

A simplified block diagram of a simultaneous wireless power and data transfer system over a single coupled-coil link for biomedical applications is illustrated in Figure 6. Both data and power are transferred over the same inductive link. The data transfer can be forward data (FD), also called downlink data, or reverse or backward data (BD), also called uplink data. In FD telemetry, data bits are sent to IMDs; in the back telemetry system, they are sent from the IMDs to external devices. The required data rate for the forward or backward telemetry system depends on the application. For instance, a high-speed FD rate is required for neural recording at rates on the order of tens of megabits per second. In contrast, artificial vision via high-density retinal implants, like neural stimulation, requires modest data rates, with BD rate requirements remaining minimal. To enable efficient, error-free data transfer over inductive coil links, modulation techniques are critical.

In the literature, various modulation techniques have been introduced, including ASK, FSK, and PSK. The above modulation techniques offer different trade-offs among complexity, bandwidth utilization, and noise resistance. FSK is useful for its resistance to amplitude variations; however, it requires a high bandwidth. ASK is less power-hungry and needs simpler circuit implementation but is more sensitive to noise. Therefore, more advanced and complex techniques, such as quadrature amplitude modulation (QAM) and orthogonal frequency division multiplexing (OFDM), have been introduced, offering high data rates and spectrum efficiency; however, these techniques are complex and consume significant power [29]. In the following section, we present downlink data communication over a single inductive coil.

**Figure 7:**
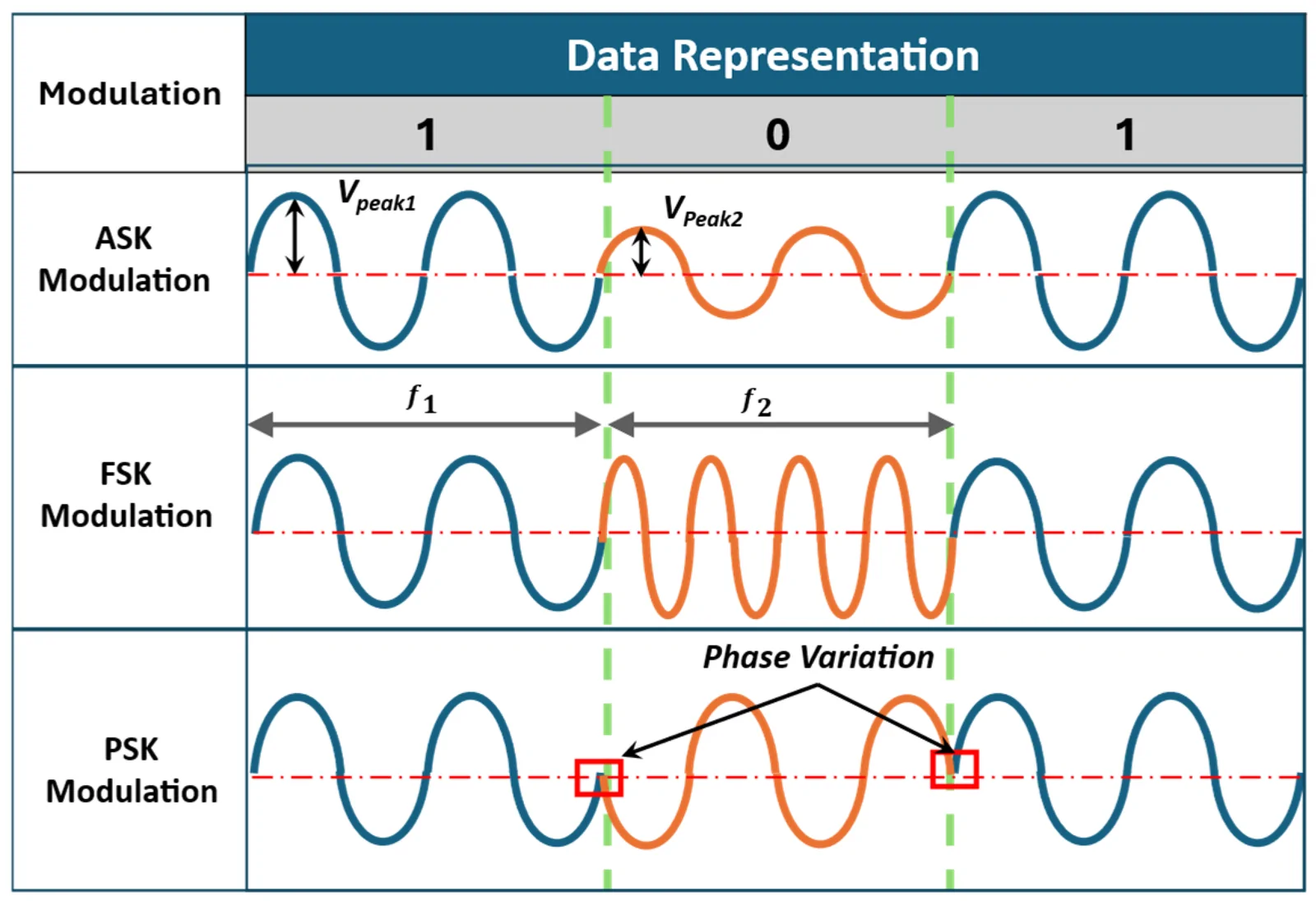
Data modulation schemes: ASK, FSK, PSK.

**Figure 8:**
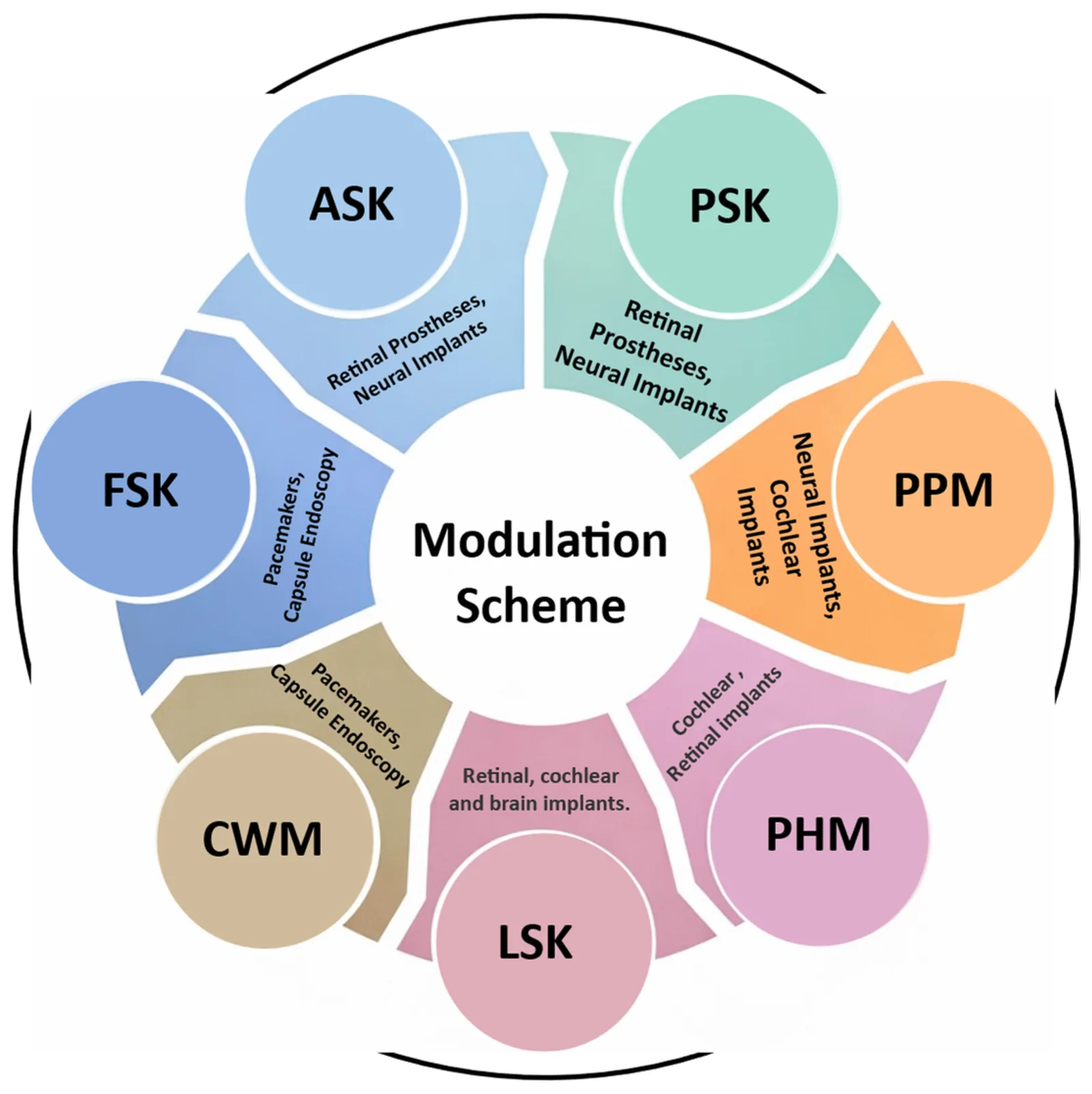
Carrier modulation types and their application.

In stimulation systems, the external unit sends commands to IMDs to determine which body part or brain region to stimulate or measure. Based on carrier modulation, these systems are classified into three categories: single-carrier, multiple-carrier, and pulse-based data telemetry systems. In this section, we focus on single-carrier modulation, which is widely used for data transmission. A single-carrier modulation scheme enables simultaneous power transfer and data communication, reducing circuit complexity and supporting compact implant integration. Figure 7 presents modulation techniques, including ASK, PSK, and FSK, in which data bits are encoded by varying the carrier’s amplitude, frequency, or phase. In pulse position modulation (PPM), the information signal is encoded in the time position of a pulse relative to a reference time instant. The positions of the pulses vary according to the instantaneous value of the message signal. In these systems, a single frequency is used for both power transfer and data modulation, reducing complexity, enabling compact designs, and improving reliability in IMD applications. The selection of modulation techniques depends on the requirements of the application. Figure 8 summarizes the key applications of ASK, PSK, FSK, and PPM schemes. For continuous power transfer, high data rate, and noise immunity, PSK and its sub-branch modulations, including quadrature PSK and quasi-coherent PSK, are preferred. However, these modulation techniques have implementation complexity and consume high power [30]. Authors in [31] have reported a data rate of 0.15 Mbps at 144 MHz. In comparison, binary phase-shift keying (BPSK) at 13.56 MHz achieves a higher data rate of 1.12 Mbps with a bit error rate (BER) of $10^{-5}$ [32]. Furthermore, an FSK-based system with the highest data rate of 2.5 Mbps and a BER of $5\times 10^{-5}$ has been proposed [33].

In this section, we focus on ASK modulation and its variants, such as on–off keying (OOK), carrier-width modulation (CWM), and quadrature-carrier width modulation (Q-CWM).

### 3.1. Amplitude Shift Keying

**Figure 9:**
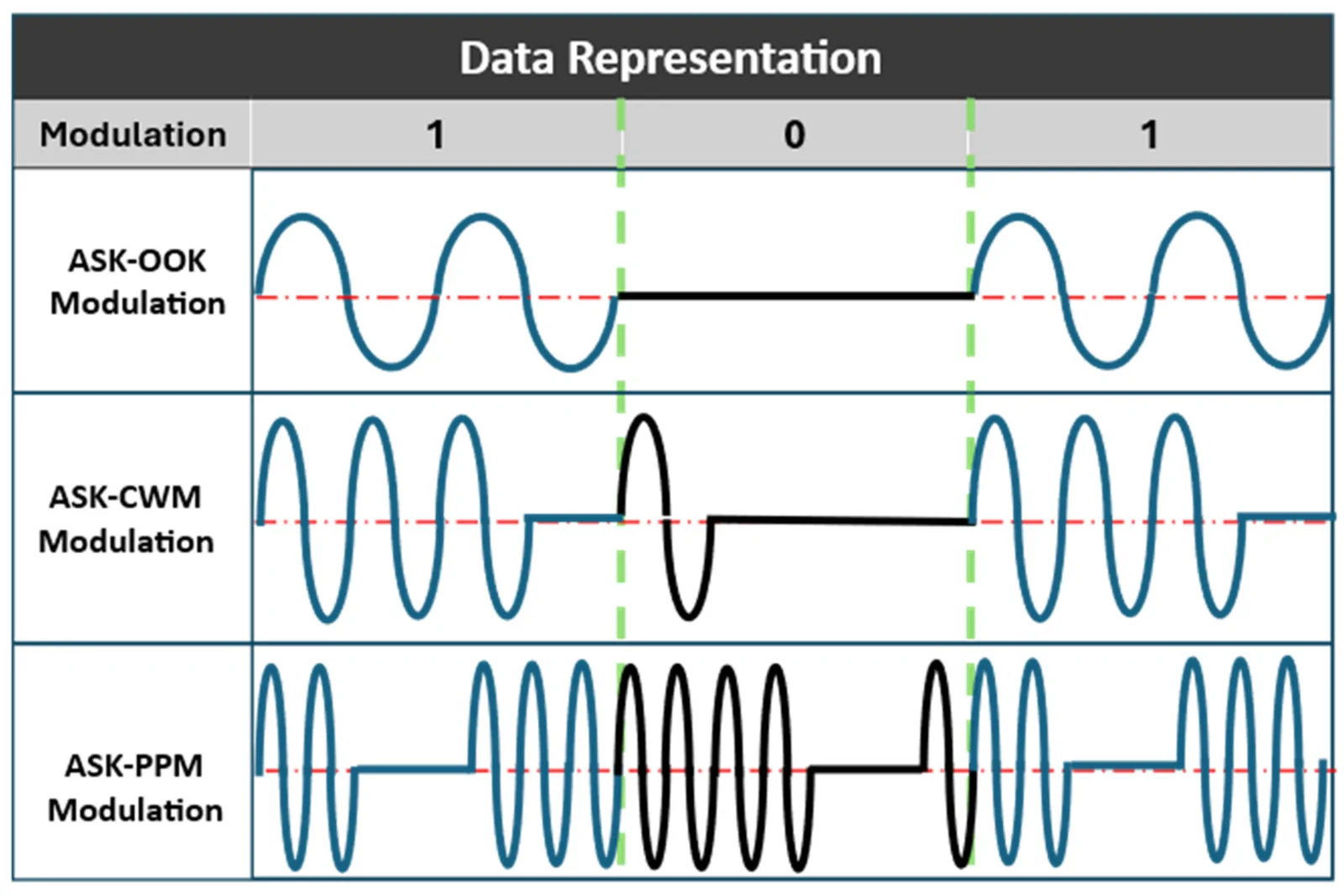
Modulation waveforms: (**Top**) ASK-OOK, (**Middle**) ASK-CWM, (**Bottom**) ASK-PPM.

**Figure 10:**
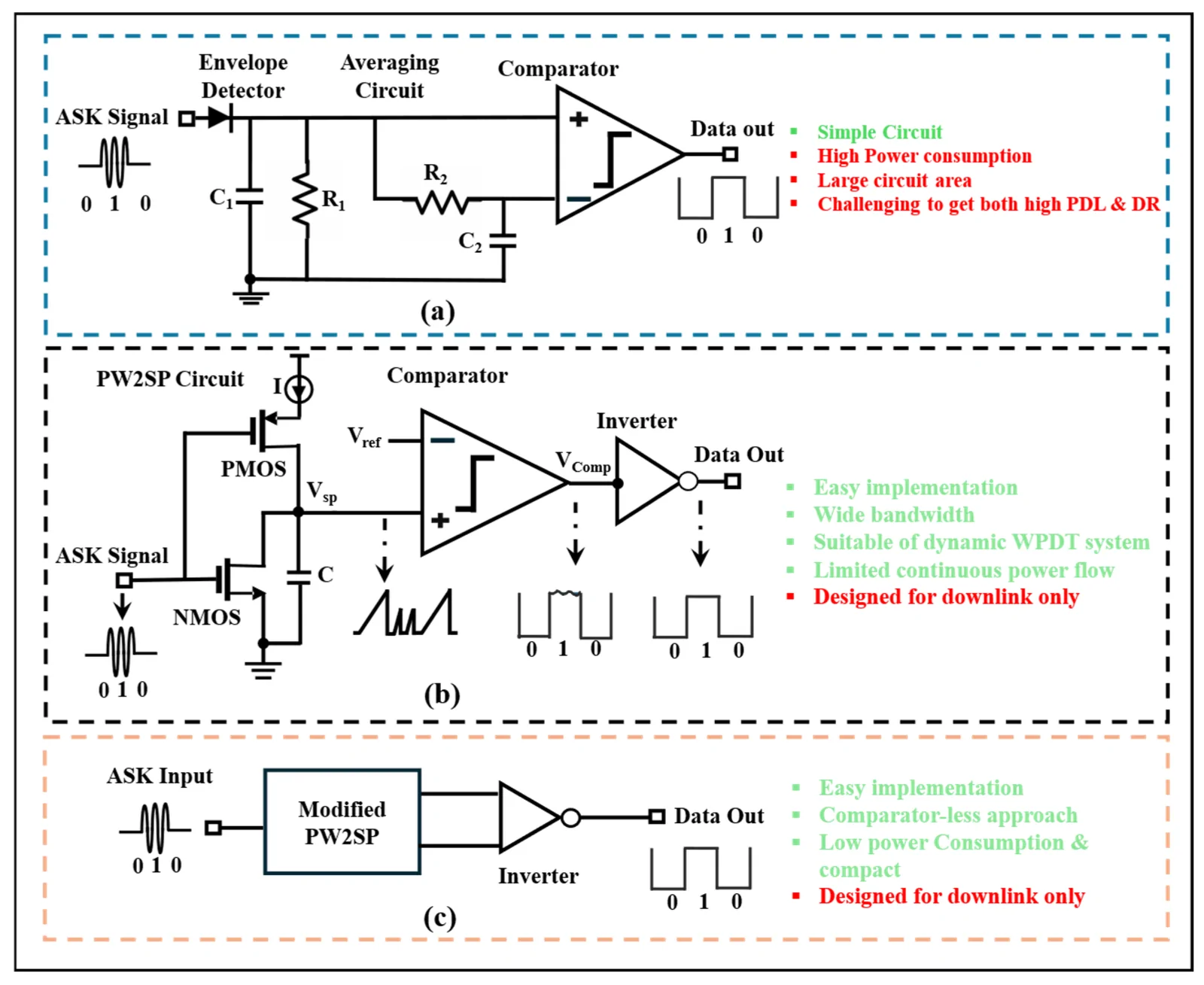
ASK demodulator circuits: (**a**) ED-based, (**b**) PW2SP circuit with comparator. Reprinted/adapted with permission from Ref. [47] (**c**) PW2SP without comparator circuit. Reproduced/adapted with permission from Ref. [48].

Among the available schemes, ASK is preferred for its low power consumption and minimal design complexity, especially in ultra-constrained implantable systems [34,35]. Its simplicity eliminates the need for complex synchronization circuitry, making it attractive for low-data-rate applications. However, ASK is inherently sensitive to variations in carrier amplitude, making it vulnerable to noise, coupling fluctuations, and external interference common in biological environments. OOK with a 100% modulation index (MI) is preferred due to improved immunity to amplitude instabilities. When transmitting a data bit “0”, no signal is transmitted, preventing continuous power delivery, as shown in Figure 9 (Top). OOK is preferred in energy-storage components to maintain power during carrier-off intervals, thereby increasing the complexity of the overall implant design. To minimize the duration of no signal transfer in OOK, different approaches have been introduced in the literature to encode data in the time domain rather than the amplitude domain, called carrier width modulation (CWM) and pulse position modulation (PPM), shown in Figure 9 (Middle) and Figure 9 (Bottom). CWM and PPM techniques have been developed in which the baseband data can be represented by varying the pulse width or position. This makes the system more resistant to noise and amplitude changes than regular ASK. Envelope detectors (EDs) are preferred in traditional ASK receivers because they are easy to implement, as shown in Figure 10a. However, the ED suffers from high power consumption due to power loss during diode switching. It also imposes severe design constraints. To accurately detect envelopes, the RC time constant must be carefully chosen in relation to the highest message frequency $\left(f_{m}\right)\;$[36]. This limits the data rate by nature. As a result, the maximum data rate achievable is limited to about one-tenth of the carrier frequency [29]. This means that envelope detectors are not good for high-speed communication. To extract the data from the ED circuit, the time constant τ should fulfill the given condition:

(5) $$\frac{1}{f_{c}}\ll \tau \ll \frac{1}{f_{m}}$$

where $f_{c}$ and $f_{m}$ are the carrier and message frequency, respectively. Due to this limitation, the circuit can only operate at a specific carrier frequency. Moreover, in the conventional WPT system, it has been observed that distance variation between the transmitter ($T_{x}$) and receiver ($R_{X}$) causes frequency splitting and a reduction in power efficiency [37,38,39]. Various solutions have been proposed to address this issue [40,41,42,43,44]. Among them, a special structure for resonators is utilized [42,43,44,45]. The adaptive matching network is the key solution approach; however, huge relay switches and additional circuitry are required. In paper [46], a variable inductor at Rx is proposed, which makes the circuit bulky. All the above solutions have been proposed to compensate for power transfer efficiently. The impact of frequency splitting in the data recovery circuit remains.

To address this issue, our group proposed a wideband ASK-OOK demodulator circuit that recovers the data using a PW2SP converter, as illustrated in Figure 10b. The circuit can support and recover data for a wide range of carrier frequencies suitable for dynamic wireless and power systems in over-coupled mode [47]. The over-coupled mode appears when the transmitter and receiver are brought close together, and the frequency is split into two frequencies such as $f_{1}$ and $f_{2}$ [48]. The operating frequency $\omega_{OP}$ can be calculated in Equation (6):

(6) $$\omega_{OP}=\left\{\begin{array}{ll} \omega_{R} & k<y\\ \omega_{R}\left(1\pm \frac{1}{2}\sqrt{\left(k^{2}-y^{2}\right)}\right) & k>y \end{array}\right.$$

where *k* is the inductive coupling coefficient of the system, and y is the loss constant. Figure 10b illustrates the wide-band data recovery circuit. In this condition, the frequency splits and generates two frequencies. The circuit achieved a 2 Mb/s data rate with a power consumption of 52.08 μW [47]. Mathematically, the exponential charging and discharging of a capacitor (C) can be expressed with Equations (7) and (8), respectively:

(7) $$V_{sp(Charge)}=V_{i}\;\left(\;1-e^{-\frac{t}{{(R}_{PMOS}\;\times \;C)}}\right)$$

(8) $$V_{sp\;(discharge)}=V_{i}\;\left(e^{-\frac{t}{{(R}_{NMOS}\;\times \;C)}}\right)$$

where $R_{PMOS}$ and $R_{NMOS}$ are the on-resistance of the PMOS and NMOS transistors, respectively. $V_{i}$ is the initial voltage. Later, we proposed a comparator-free ASK-OOK demodulator circuit to reduce the chip size and power consumption [35]. Figure 10c shows the comparator-free ASK-OOK demodulator circuit. The data can be recovered by passing the input-modulated signal through a modified PW2SP circuit for conversion, followed by an inverter circuit. The modulated signal is applied to the sawtooth peak. This approach provides a suitable balanced trade-off between size, power, and data rate, making it suitable for biomedical implants. A key drawback of ASK and its derivative modulation schemes is their limited ability to handle high data rates. In real-world applications, the maximum bandwidth is usually only about one-tenth of the carrier frequency [49]. This limitation arises from two principal factors when these methods are utilized over a power carrier. A high Q factor is good for maximizing PTE, but it also reduces the available bandwidth because Q and bandwidth are inversely related. Because of this, the system bandwidth must remain low enough to enable efficient power transfer, which directly limits the maximum achievable data rate. The author in [50] developed a suspended-carrier technique to overcome the Q-factor bandwidth limit. This made it possible to relax this trade-off and improve data rate performance. The suspended-carrier modulation scheme is similar to OOK but differs significantly in system architecture and modulation timing. In conventional OOK, modulation is achieved by directly switching the DC supply or the AC input of the power amplifier without requiring strict timing [51,52,53,54]. In comparison, suspended-carrier modulation uses synchronized switching that occurs when the primary coil current crosses zero. To do this, a special modulation circuit is needed.

From a performance standpoint, suspended-carrier modulation mitigates a significant drawback of traditional ASK-based systems. Its bandwidth is largely unaffected by the quality factor of the secondary resonant circuit [24,55]. Despite these advantages, suspended-carrier modulation introduces several practical limitations. Power delivery to the load occurs only during the transmission of logic “1” bits, resulting in a reduced average power transfer of approximately 50% under equal conditions and equal probabilities of “1” and “0” bits.

### 3.2. Carrier Width Modulation (CWM)

There are several downlink or uplink modulation schemes that have been proposed for data transfer over inductive links [56,57,58,59,60,61,62,63,64,65,66,67,68,69,70,71,72,73,74]. Among them, carrier width modulation (CWM), a more sophisticated data modulation method for forward data communication, has been introduced. This technique works like traditional suspended-carrier modulation, in which the carrier cutoff occurs when the primary-coil current drops to zero. The ON duration, on the other hand, can last for any number of cycles, which are defined as (n − 2) to express data bit “1” and (n − 1) for bit “0”, where n is the number of carrier cycles in a single data bit interval and can be any integer value greater than or equal to 3. The CWM method addresses weaknesses of traditional modulation methods, such as (1) limited data throughput due to demodulator constraints, (2) high sensitivity to amplitude variations, and (3) discontinuities in wireless power transfer [23,56,57].

**Figure 11:**
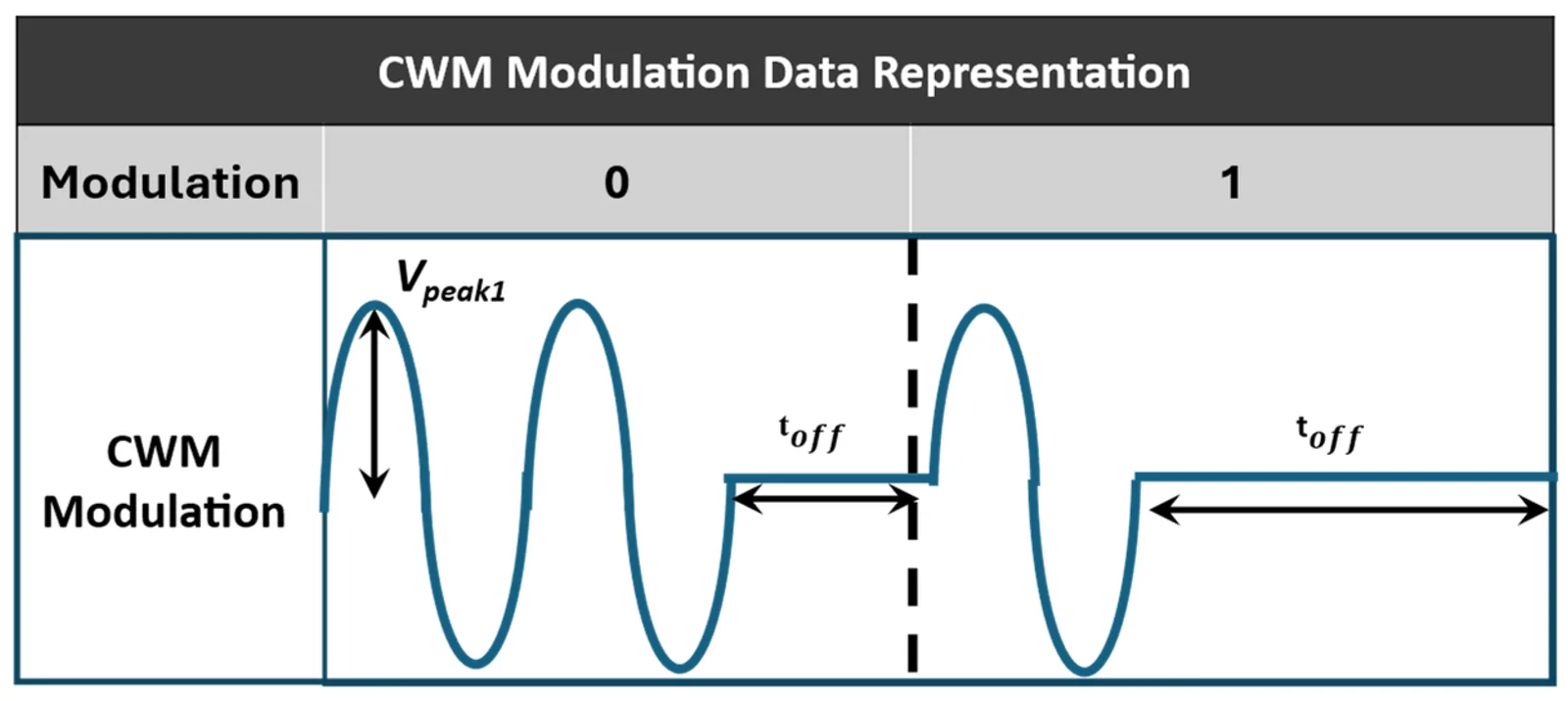
Typical CWM data representation.

Figure 11 shows the data representation of CWM and indicates that toff occurs when the voltage remains constant. A longer toff means the absence of the carrier. The number of cycles for the data representation can be chosen based on application requirements. However, the maximum data rate using CWM is one-third of the carrier frequency. For example, if the carrier is 27 MHz, which is widely used in the ISM band. The maximum data rate is achieved at 9.04 Mb/s. The powering index (PI) is a key metric in CWM that quantifies the effectiveness of power transfer during simultaneous data communication, as given in Equation (9):

(9) $$PI=1-\frac{3}{2n}$$

where *n* represents the number of cycles per data bit. The n parameter is important to decide between a high data rate and PTE. As the small value of n increases, the data rate increases but reduces PI, potentially compromising power stability, whereas higher values of n improve power delivery at the cost of reduced data rate.

**Figure 12:**
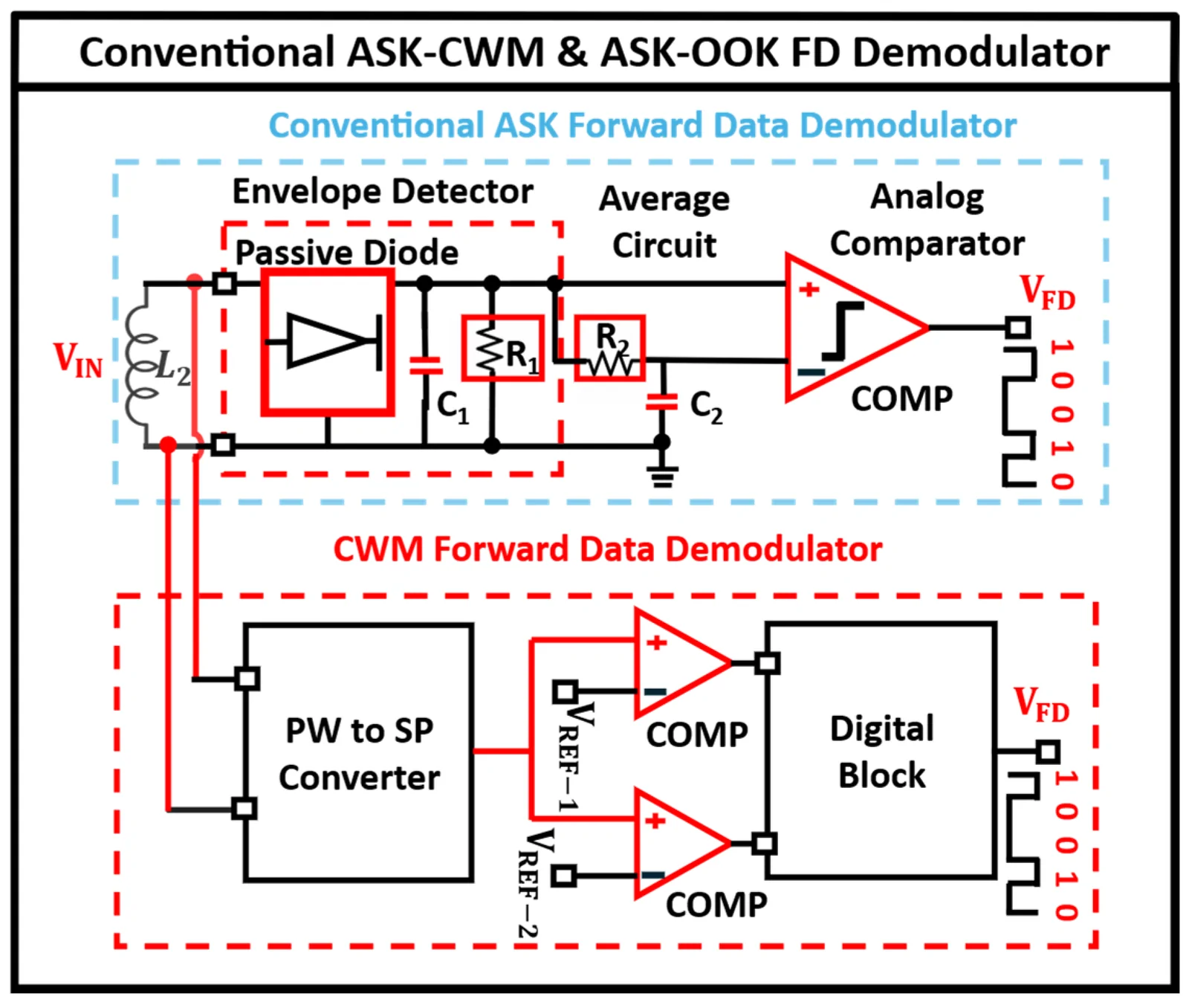
Conventional ASK forward data recovery circuit and CWM forward data recovery circuit.

Therefore, the powering index is a critical design parameter for optimizing system performance, ensuring reliable operation, and maintaining a sufficient energy supply during wireless power and data transfer. As n decreases, the data rate increases while the powering index decreases. Specifically, for n = 100, the system achieves a data rate of 0.2712 Mb/s with a high-power index of 98.5%. When n is reduced to 20, the data rate increases to 1.356 Mbit/s, while the power index decreases slightly to 92.5%. A further reduction in n results in a significantly higher data rate of 5.424 Mb/s. This is the trade-off between data rate and power transfer efficiency in CWM systems. The CWM technique was utilized to achieve high data rates and low power consumption. This technique also uses a pulse width-to-sawtooth peak (PW2SP) circuit, unlike conventional envelope detectors, which use RC and averaging circuits, to extract the data (VFD), as shown in Figure 12. The output of the PW2SP circuit is fed to a comparator circuit, followed by a digital circuit that includes a delay circuit and a mux to recover the clock and data.

#### Quad-Level CWM

**Figure 13:**
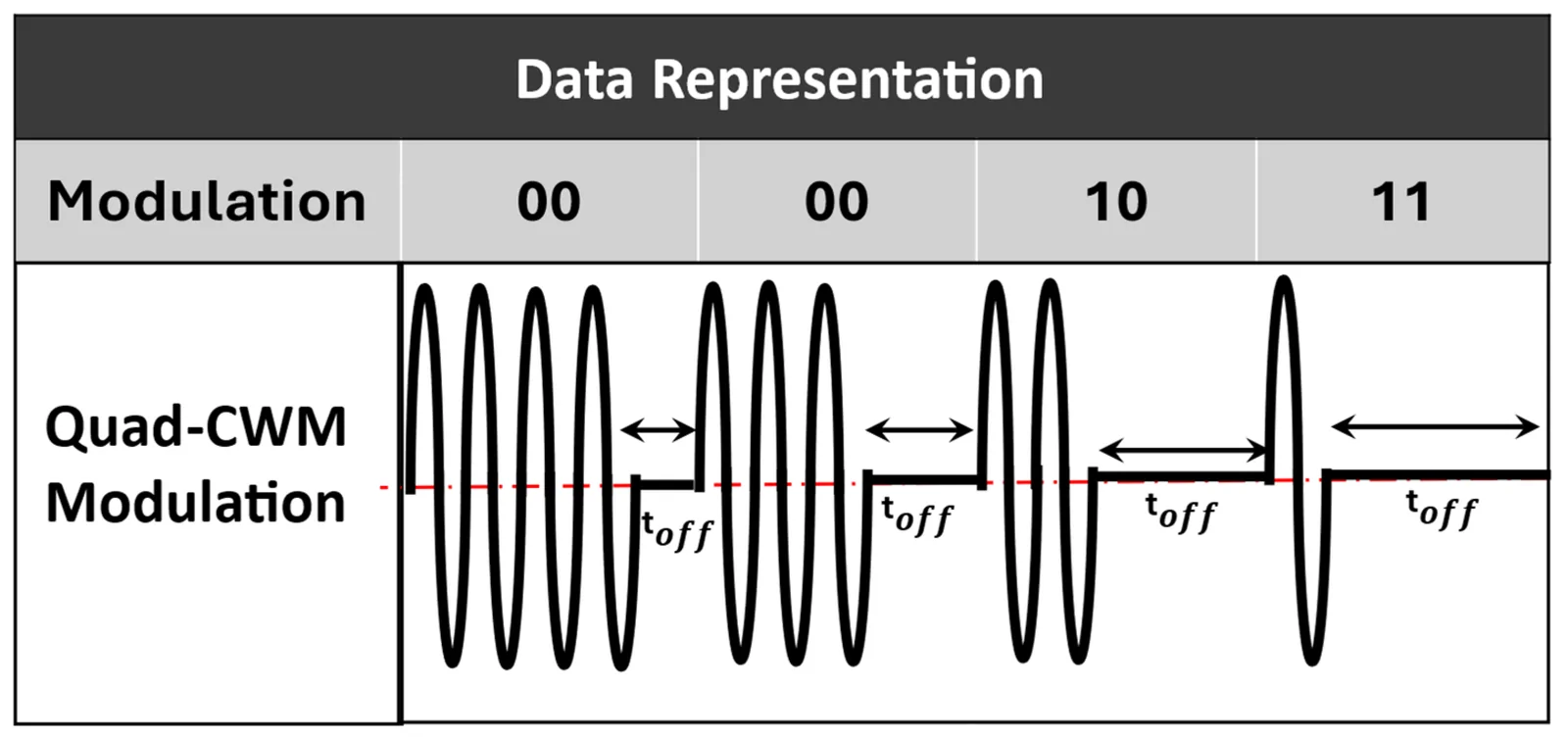
Quad-level CWM modulated signal.

The quad-level CWM technique was also proposed and implemented to achieve high data rates and low power consumption. Compared with ASK, FSK, and PSK, QCWM offers several advantages, including a compact footprint, low power consumption, and a wide frequency range. By encoding two bits per symbol through four different duty-cycle levels, QCWM can send more data than regular CWM, as shown in Figure 13. Two methods have been proposed for extracting data from a quad-level CWM signal. Both methods are explained in the following section.

(a) Pulse width-to-sawtooth peak (PW2SP) circuit quad-level CWM

**Figure 14:**
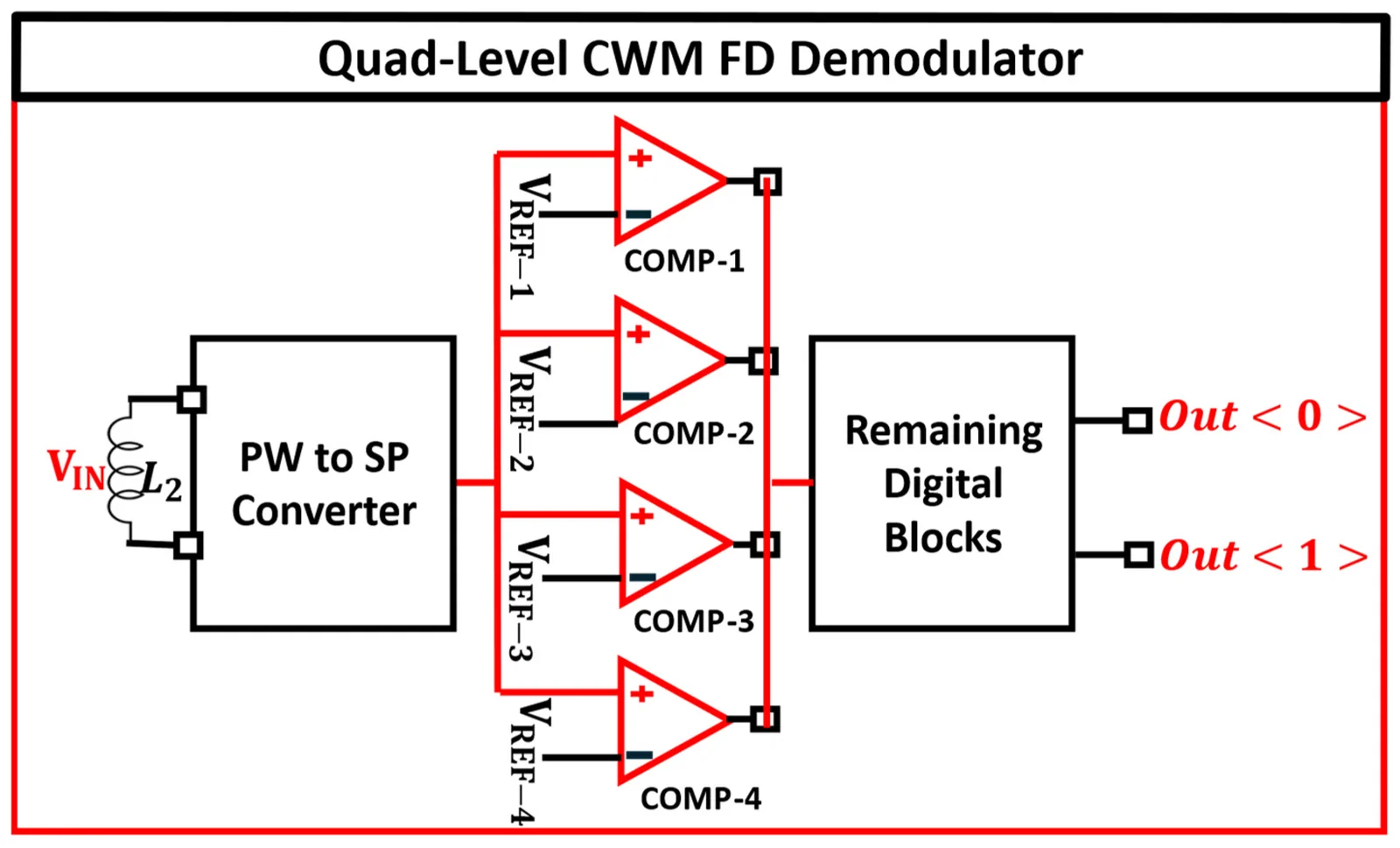
Conventional forward data recovery circuit using quad-level CWM modulation.

Similarly to CWM, this technique also uses pulse width to convert a sawtooth peak into a PW2SP. In addition, more comparators and logic circuits are employed to extract the data, as shown in Figure 14. Both CWM and QCWM-based PW2SP data recovery circuits are energy-efficient, consuming very little power while still supporting high data rates. CWM also has the benefit of being able to change the data rate and the powering index. However, the operating frequency range is still limited.

(b) Counter-based quad-level CWM demodulator

**Figure 15:**
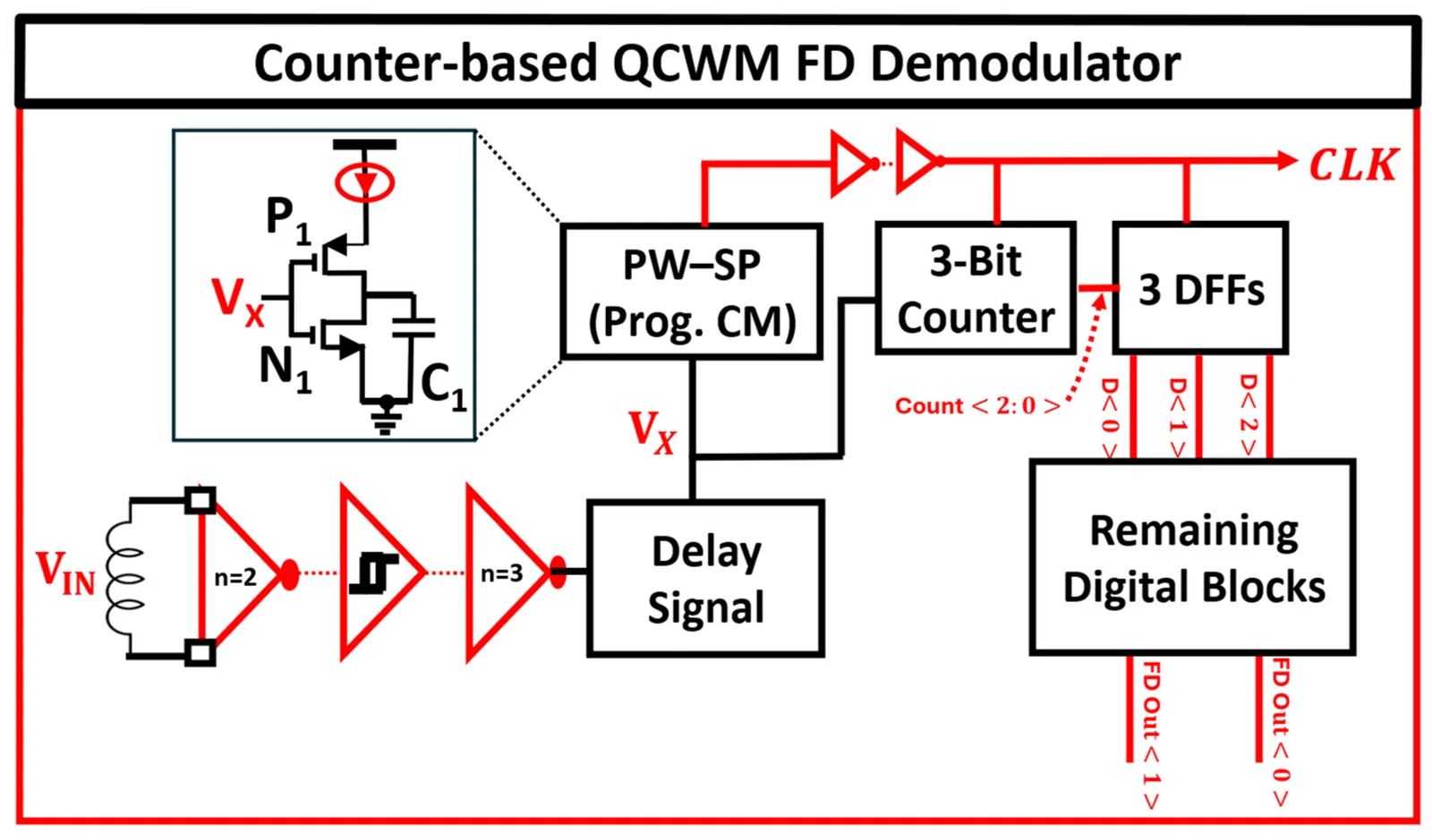
Counter-based quad-level CWM demodulator circuit for clock and data recovery circuit.

Recently, our group presented a counter-based CMOS QCWM forward data demodulator designed to operate over a wide range of carrier frequencies [58]. The conventional approaches illustrated in Figure 14 rely on a PW2SP converter combined with a series of comparators requiring more than one reference voltage, which constrains the operating range due to the current-starved behavior of the PW2SP stage. To overcome this limitation, we propose a modified PW2SP architecture incorporating a programmable current mirror controlled by a 3-bit counter, enabling tunable current operation while eliminating the need for an external reference voltage, as shown in Figure 15.

The proposed demodulator, designed in a 180 nm CMOS process, operates over a wide frequency range from 1 MHz to 20 MHz and supports data rates from 400 kb/s to 8 Mb/s. At 20 MHz, the circuit consumes 209 µW. The circuit provides a flexible and scalable solution for conventional QCWM demodulators. It can be utilized for applications requiring a wideband carrier frequency range with moderate power consumption. In the conventional approach, as the frequency increases, the capacitor charges more quickly. As a result, the peak voltage $\left(V_{peak}\right)$ decreases at higher frequencies, which requires an external reference voltage ($V_{ref})$ to be lowered. The achievable data rate in this architecture is limited by $V_{ref}$, as it is directly proportional to the carrier frequency. A limitation arises from the constant current used to charge the capacitor. At higher carrier frequencies, a high current is required to adequately charge the capacitor.

Mathematically, it can be described in Equations (10) and (11). Overall, both the upper and lower limits of the carrier frequency are constrained by $V_{ref}$ reaching the supply and ground rails.

(10) $$V_{\mathrm{peak}\;}=\frac{I\times t_{off}}{C}$$

(11) $$V_{\mathrm{peak}\;}=\frac{I}{C\times f}$$

**Figure 16:**
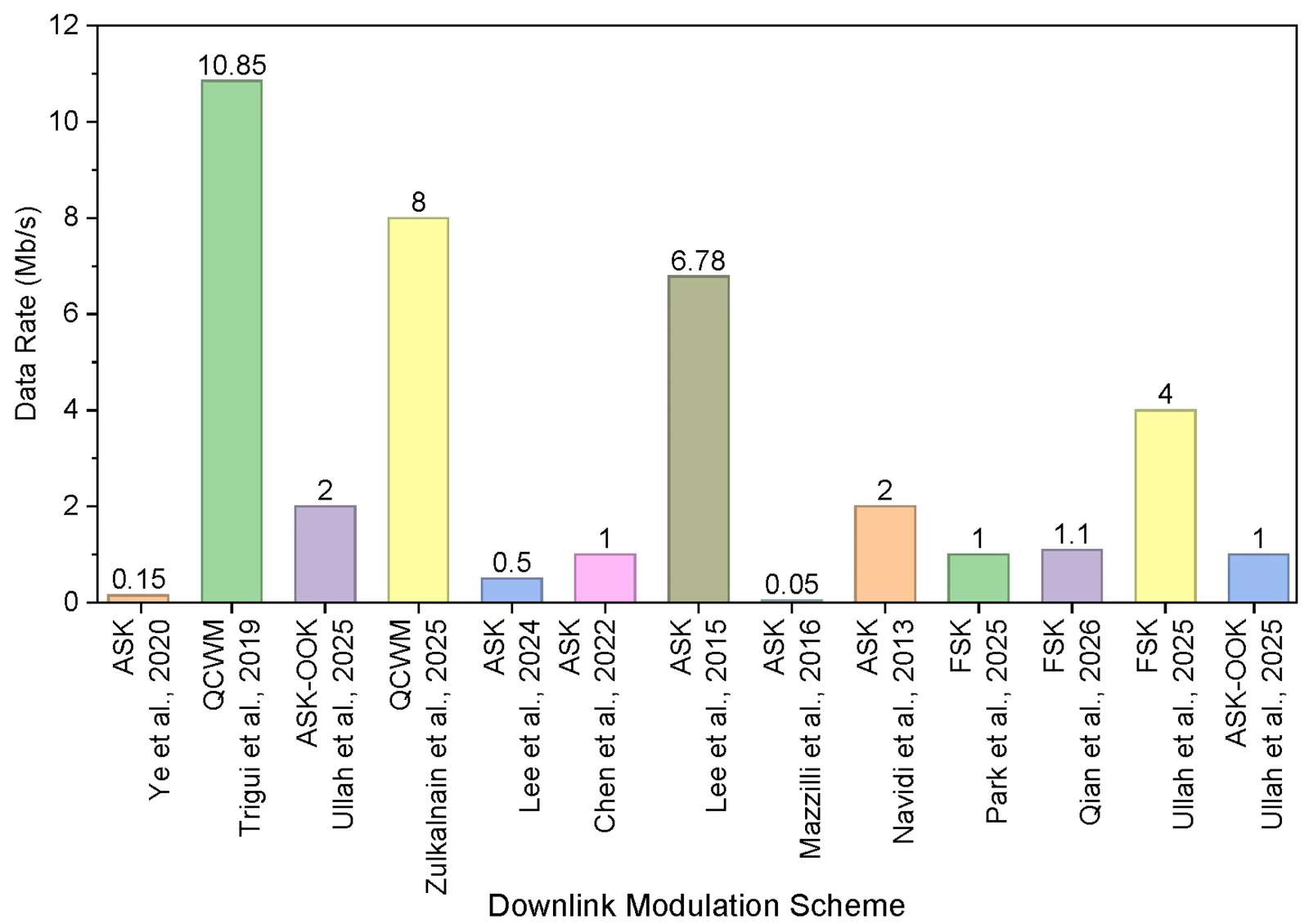
Literature comparison of downlink modulation schemes reported in [31,36,48,58,75,77,78,79,80,81,82,83,84,85] in terms of data rate.

**Figure 17:**
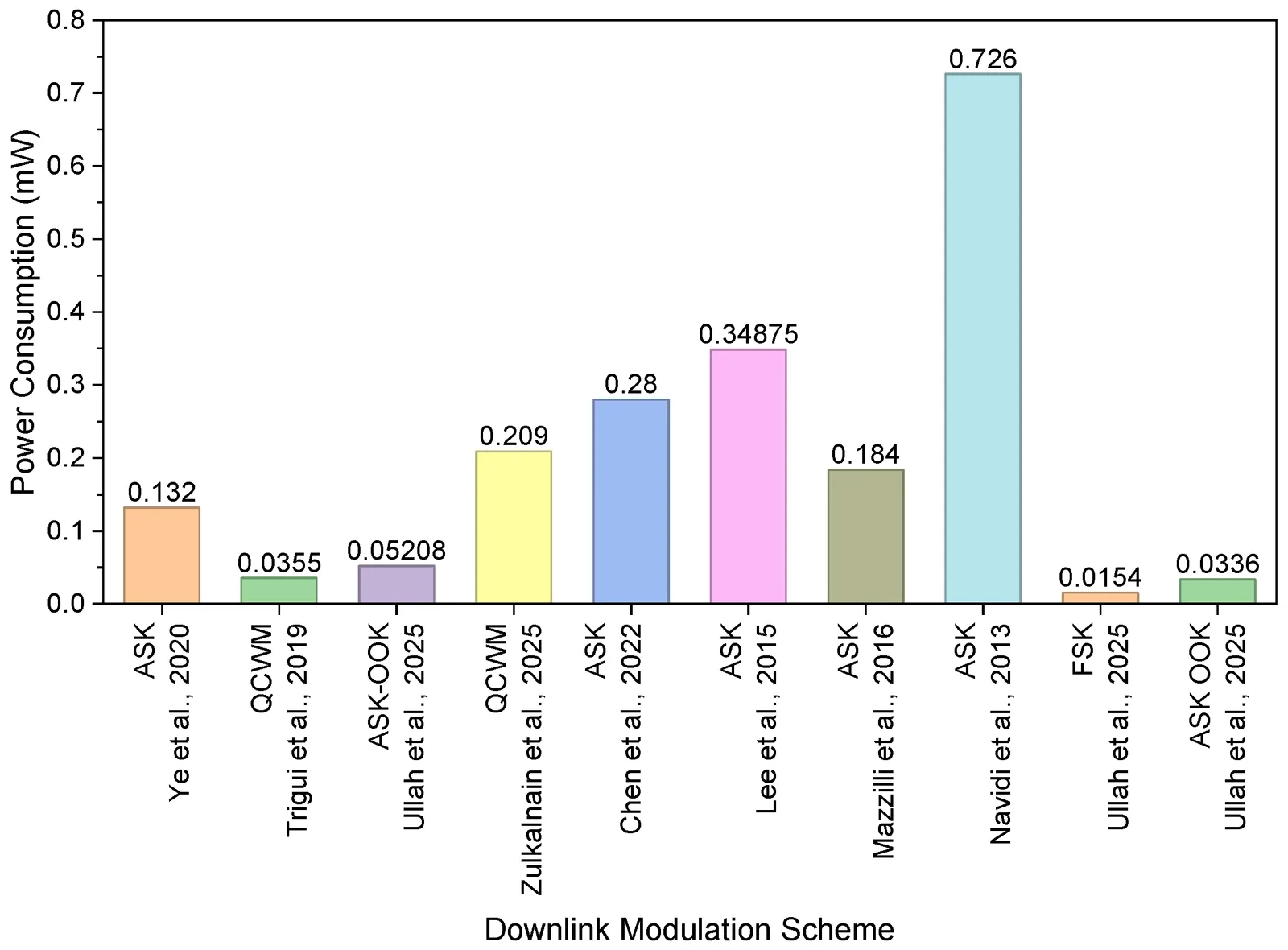
Literature comparison of downlink modulation schemes with respect to power consumption reported in [31,36,48,58,75,77,78,79,80,81,82,83,84,85].

By designing a programmable current mirror, we have achieved a maximum frequency of 20 MHz and a data rate of 8 Mb/s, as shown in Figure 16, in line with another reported research study. It is important to note that downlink modulation approaches can only support transferring data from external units to IMDs. Table 1 summarizes the carrier frequency, data rate, power consumption, BER, and silicon area of various downlink modulation approaches. According to the reported work, QCWM provides a high data rate, while ASK-OOK can support data recovery for a wide range of carrier frequencies [36,48,58]. However, BER and power delivery with an inductive coil system remain. Figure 16 shows the achievable data rate for downlink data transfer using the downlink modulation scheme, and Figure 17 illustrates the power consumption of various data recovery systems for downlink data transfer. It is noted that QCWM provides data rates of 10.85 Mb/s and 8 Mb/s while consuming 0.0355 mW and 0.209 mW, respectively.

**Table 1:** Benchmarking of recent downlink data modulation methods.

Work	Year	Tech (nm)	Link Architecture	Freq. (MHz)	Modulation	Data Rate (Mb/s)	Power Consumption (mW)	BER	Area (mm^2^)
[31]	2020	65	Single	13.56	ASK	0.15	0.132	<10^−3^	-
[36]	2019	130	Single	31	QCWM	10.85	0.0355	-	3.25
[47]	2026	180	Single	5–150	ASK-OOK	2	0.05208	-	0.00244
[58]	2025	180	Single	20	QCWM	8	0.209	-	1.944
[75]	2024	250	Single	2	ASK	0.5	-	<10^−4^	0.023
[76]	2025	180	Single	6.8–7.2	FSK	1	-	<10^−7^	-
[77]	2022	180	Multiple	13.56	ASK	1	0.28	-	510
[78]	2015	180	Single	13.56	ASK	6.78	0.34875	-	80
[79]	2016	180	Ultrasonic	1	ASK	0.05	0.184	-	-
[80]	2013	130	Single	10	ASK	2	0.726	-	16.8
[81]	2025	180-BCD	Single	-	FSK	1	-		-
[82]	2026	180	Single	-	FSK	1.1	-		-
[83]	2025	180	Single	4, 8	FSK	4	0.0154	<10^−8^	0.00274
[84]	2021	180	Single	150	ASK-OOK	1	0.0336		1.08
[85]	2021	180 BCD	Single	-	FSK	2.5	-	<10^−6^	-

### 3.3. Frequency Shift Keying

In FSK, the data bits are encoded by changing the carrier frequency. Both data and power can be transferred using the same inductive coils. Unlike ASK, the FSK modulation technique provides better resilience to noise, which improves signal integration and enhances communication reliability [76,81,82,83]. However, FSK requires high bandwidth for passing the modulated frequency over an inductive link [29]. Existing FSK modulation-based systems utilize multiple carrier cycles, such as four cycles and two cycles for data bits one and zero, respectively. The enhanced splitting-based design using FSK modulation was proposed with dynamic link load isolation, enabling a data rate of 1 Mbps and power transfer of 43.4 mW. A dual-mode operation design for power and data transfer is proposed with large frequency separation. The design supports a 1.1 Mbps data rate with a power delivery capability of 148 mW [82]. To enhance the data rate to carrier frequency (DRCF) ratio, an FSK-based clock and data recovery circuit is proposed that utilizes only two cycles and one cycle of the carrier signal to encode the data. The reported design supports a data rate of 4 Mb/s [83]. In order to meet the requirements of high power delivery and high data rates, a frequency splitting-based FSK WPDT system is proposed. The system can deliver power of 115 mW with a data transfer rate of 2.5 Mbps using a single inductive link for next-generation cochlear implants.

### 3.4. Alternative Modulation Techniques

There are several alternative modulation techniques to transfer power and data, including PSK, pulse duration modulation (PDM), and pulse harmonic modulation (PHM). PSK is a single-carrier modulation technique that represents the data signal by varying its phase while maintaining its amplitude and frequency constant. In comparison with ASK, it provides robustness to noise and improves communication reliability. However, an intelligence system is required to extract the data from the receiver, making it a complex approach.

In PDM, the data bits are encoded by changing the pulse width or duration of the signal. Compared with PSK, the architecture is simple, but the achievable data rate is limited by the pulse time requirement [51]. Authors in [34,35] presented PHM, which is a pulse-based modulation technique in which the data can be transferred in the form of a pulse signal. PHD can support data rates of up to 20 Mbps and 10.2 Mbps [69,70]. Both approaches are carrier-less and improve the BW; however, they can only support data transfer over a single inductive coil, as they cannot efficiently transfer power, and increasing the frequency of the carrier signal can cause potential tissue damage. Therefore, to transfer power, a dedicated link is required that increases the size of the design; therefore, it is rarely used for a simultaneous WPDT system.

## 4. Uplink Data Communication over a Single Inductive Link

**Figure 18:**
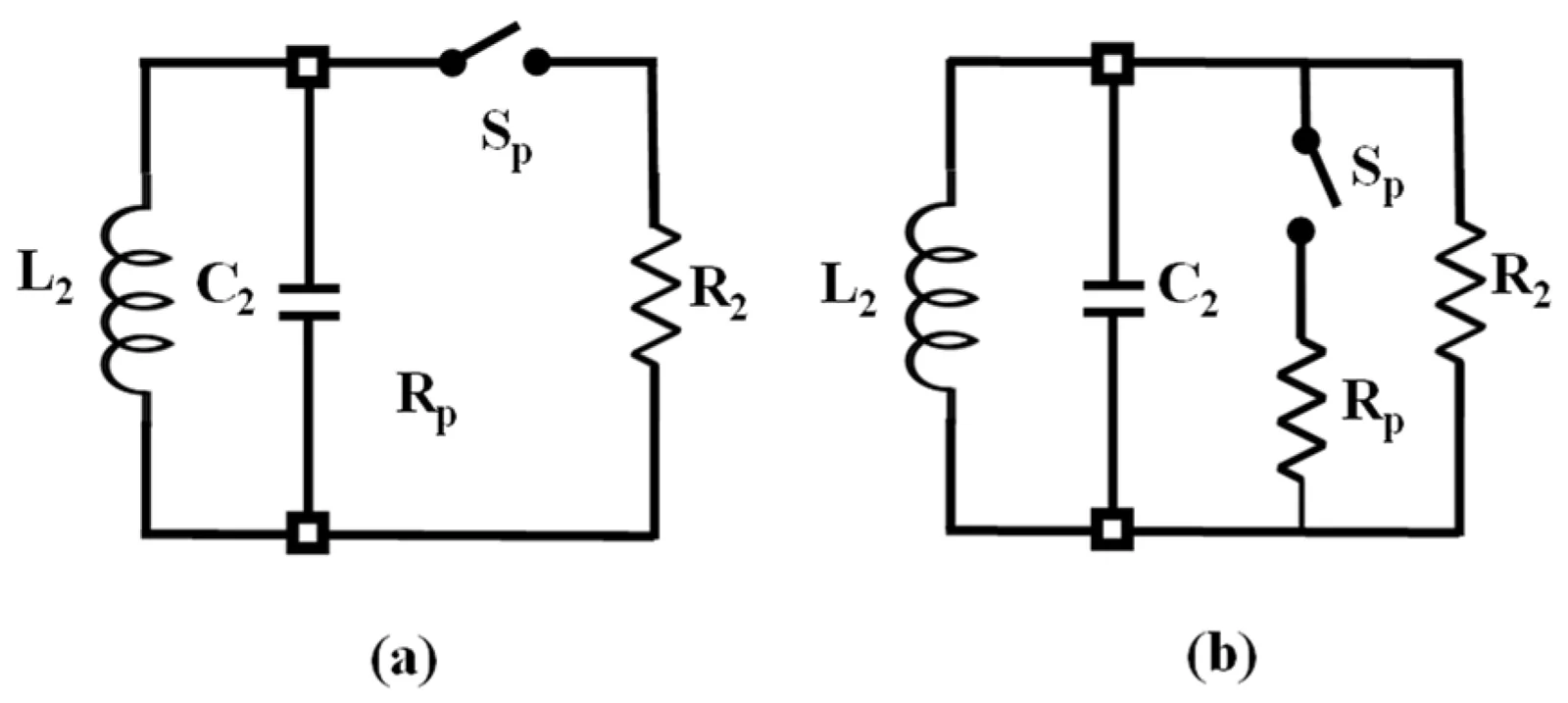
LSK modulation working principle using the resistive modulation approach for (**a**) series and (**b**) parallel resistance.

Uplink data communication, also called back telemetry, allows an internal or remote device to send data back to the external system. It is very important to retrieve sensor values, device status, or diagnostic information. There are two main ways to send data back to an external unit, namely, passive telemetry and active telemetry. Each method has its own merits and limitations. In active back-telemetry, IMDs produce their own magnetic field to transmit data and require a dedicated power source, such as an onboard battery [59] or energy harvested from a separate inductive link, along with additional circuitry, including oscillators and dedicated transmitting coils or antennas. These systems can support ASK, FSK, and PSK, and their variants can achieve higher bandwidth, extended frequency range, and high data rates, even for deeply implanted devices. Furthermore, active systems exhibit improved tolerance to coil misalignment and variations in separation distance. However, these advantages come with the drawbacks of higher power consumption and more complex circuits, which can limit their use in implantable applications. Passive back-telemetry, on the other hand, uses a magnetic field generated outside the system to transmit data by varying the load on the inductive link. This means that the IMD does not need any extra transmitters or power sources and therefore uses less power and is compact. For simultaneous wireless power and data transfer over a single inductive link, the modulation techniques such as LSK, passive PSK, and cyclic on–off keying (COOK) are widely adopted in passive back-telemetry systems. Among them, LSK, also referred to as impedance modulation, reflectance modulation, or absorption modulation, is the most widely adopted approach [51] and extensively utilized in applications such as RF-ID systems, wireless sensing platforms, and IMDs. In LSK, data transmission is achieved by modulating the load impedance of the secondary-side circuit in accordance with digital information, as illustrated in Figure 18a,b. The resistive load modulation can be implemented in either series or parallel configurations. In the series configuration, the load transitions between a finite resistance R2 and a high-impedance (open-circuit) state, requiring R2 to be sufficiently small to ensure distinguishable modulation levels. In contrast, the parallel configuration toggles the load between R2 and a low-impedance (short-circuit) condition, making it more suitable to employ either configuration or a hybrid approach [60].

**Figure 19:**
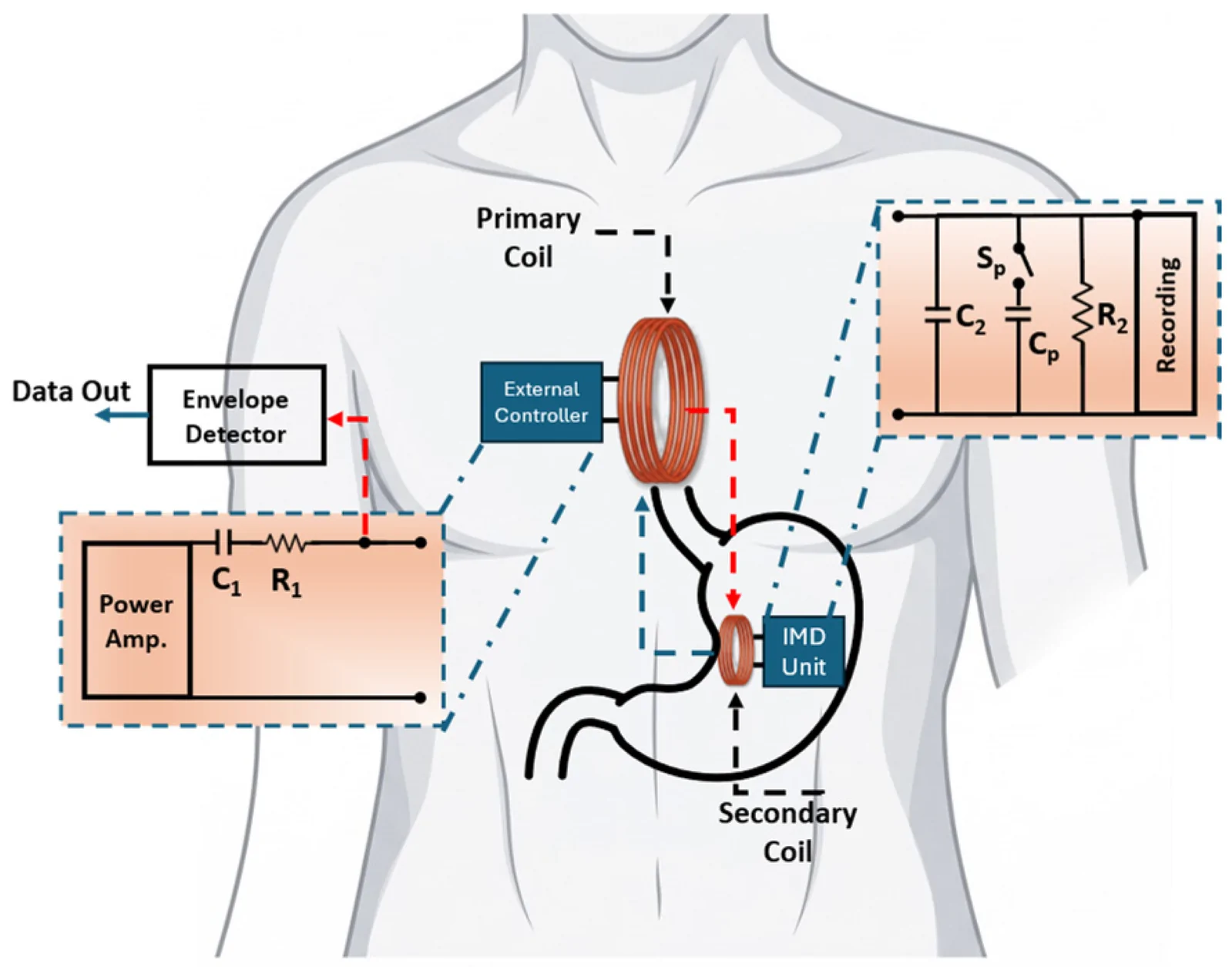
Block diagram of the concurrent wireless power transfer and gastric bioelectrical activity acquisition system employing LSK-based telemetry.

Passive back-telemetry, on the other hand, mainly achieves capacitive load modulation through variations in capacitive loads, as shown in Figure 19. It operates by perturbing the larger load values. Depending on the load characteristics, an optimal design may require impedance phase and detuning the secondary resonant frequency, as in Equation (12):

(12) $$f_{r2}=\frac{1}{2\pi \sqrt{L_{2}C_{2}}}$$

**Figure 20:**
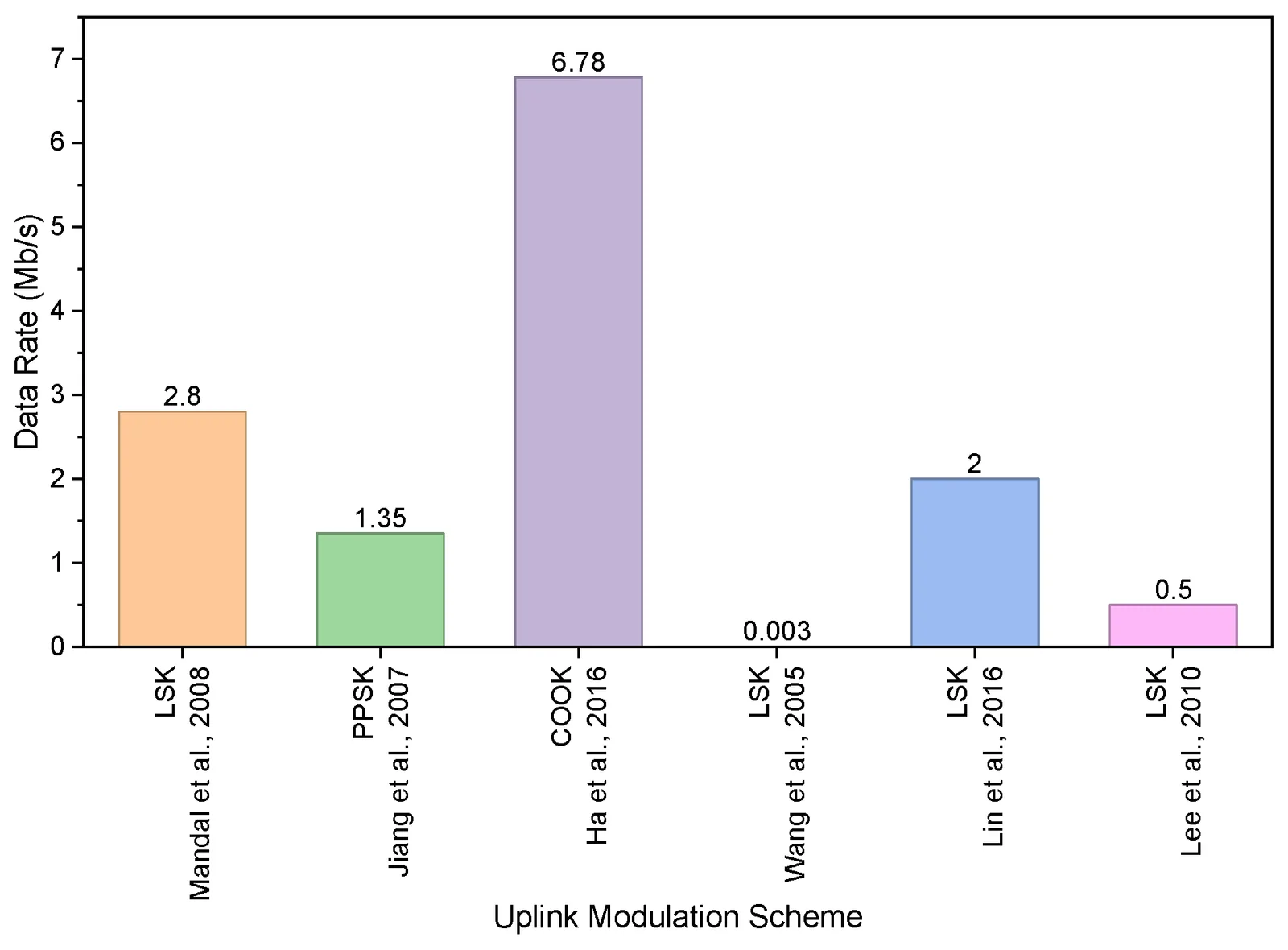
Comparison of reported data rates for different uplink modulation schemes in duplex systems [20,50,61,86,87,88].

Unlike resistive modulation, capacitive switching can bring larger variations. The LSK-based schemes exhibit inherent limitations, such as in forward-link modulation, where the achievable data rate is limited by the Q of the inductive link; high-Q operation favors efficient power transfer but restricts bandwidth. Moreover, the introduction of an additional modulation load leads to impedance mismatch, reducing PTE and the transferred energy away from the primary load during data transmission. To address these limitations, a passive PSK is introduced to enable simultaneous power and data transfer over a single inductive link without introducing additional loading, thereby mitigating the efficiency degradation associated with conventional LSK. Passive phase-shift keying (PPSK) and COOK are also widely used for simultaneous wireless power and data transfer [20,60]. PPSK holds the current near its peak during switching, forcing a phase reversal in both current and voltage. This phase shift appears as an amplitude, with switching representing logic “1” and no switching representing “0.” The data rate depends on the transient response of the resonant circuit, with faster settling enabling higher rates. At 13.56 MHz, PPSK achieves up to 1.35 Mb/s with only about a 6% reduction in PTE. However, PPSK is limited by a trade-off between PTE and data rate, as the circuit requires recovery time after each bit. High data rates require low-Q operation, which reduces efficiency and typically limits the data rate to about one-tenth of the carrier frequency, while phase switching also introduces additional losses. COOK mitigates this trade-off by using a high-Q secondary tank to enable faster recovery, supporting both high efficiency and high data rates. Figure 20 shows the data rate and area of different uplink data recovery circuits reported in the literature. Authors in [61] proposed two COOK schemes, such as 1-bit encoding over four carrier cycles and 2-bit encoding within the same interval by shifting a single-cycle shorting event. At 13.56 MHz, COOK achieves up to 6.78 Mb/s with a BER over a 1 cm link and a maximum PTE of 89.2% with a 100 Ω load, as shown in Figure 20. Despite these developments, there are still some practical challenges, such as envelope detection, which might slow down high-speed performance. The size, spacing, and alignment of the coils also affect how well the system works. Larger coils and shorter coil spacing improve coupling, BER, and delivered power, while misalignment degrades performance.

As shown in Figure 20, the maximum data rate is achieved using the COOK modulation scheme for the uplink data rate and consumes 64 µW.

Table 2 presents a benchmark of recent uplink data modulation approaches for transferring power and data over inductive coils. Among the reported approaches, the COOK-based modulation scheme achieved the highest data rate and higher power-delivery capability. All these uplink modulation techniques only support data transfer from IMDs to external units.

## 5. Full-Duplex Data Communication System

To enable simultaneous downlink and uplink communication over a shared inductive link without mutual interference, two transmission modes are employed, called full-duplex and half-duplex modes [62]. Full-duplex operation is feasible when the uplink and downlink modulations are orthogonal, such as when combining FSK or PSK for the downlink with LSK for the uplink [63,64]. However, when similar modulation schemes, such as ASK-based techniques, are employed in both directions, interference becomes unavoidable, necessitating half-duplex communication, typically implemented via time-division duplexing [20,62,65]. To address the limitations of conventional WPDT systems, a single-link full-duplex architecture operating at 40.68 MHz is proposed in [66], achieving over 50 mW power delivery and a data rate exceeding 20 Mb/s without additional hardware. The system employs resonance-length keying (RLK) for the uplink and instant-carrier-suspension keying (Instant-CSK) for the downlink, thereby removing the quality-factor limitations of ASK. These techniques operate simultaneously without interference, overcoming the drawbacks of traditional ASK and LSK schemes and enabling efficient, high-data-rate bidirectional communication and compact implantable devices.

**Figure 21:**
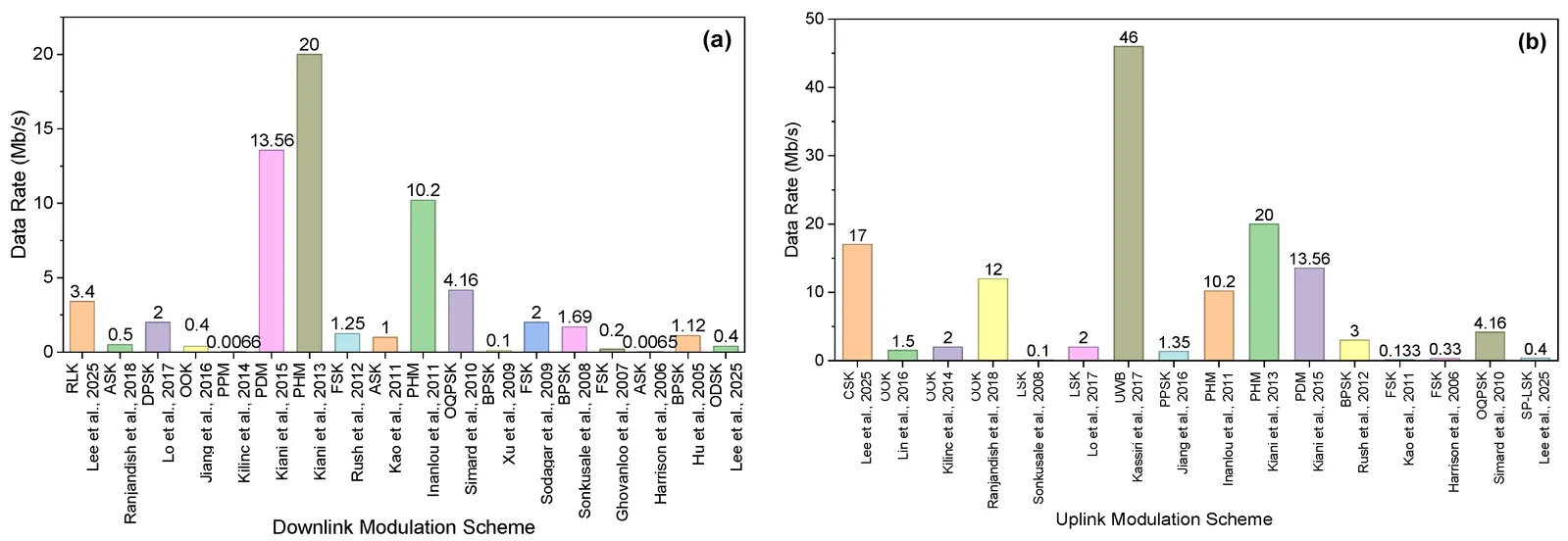
Literature comparison of full-duplex systems reported in [20,32,51,64,66,67,68,69,70,71,87,88,89,90,91,92,93,94,95,96,97]: (**a**) data rate for downlink, (**b**) data rate for uplink.

**Figure 22:**
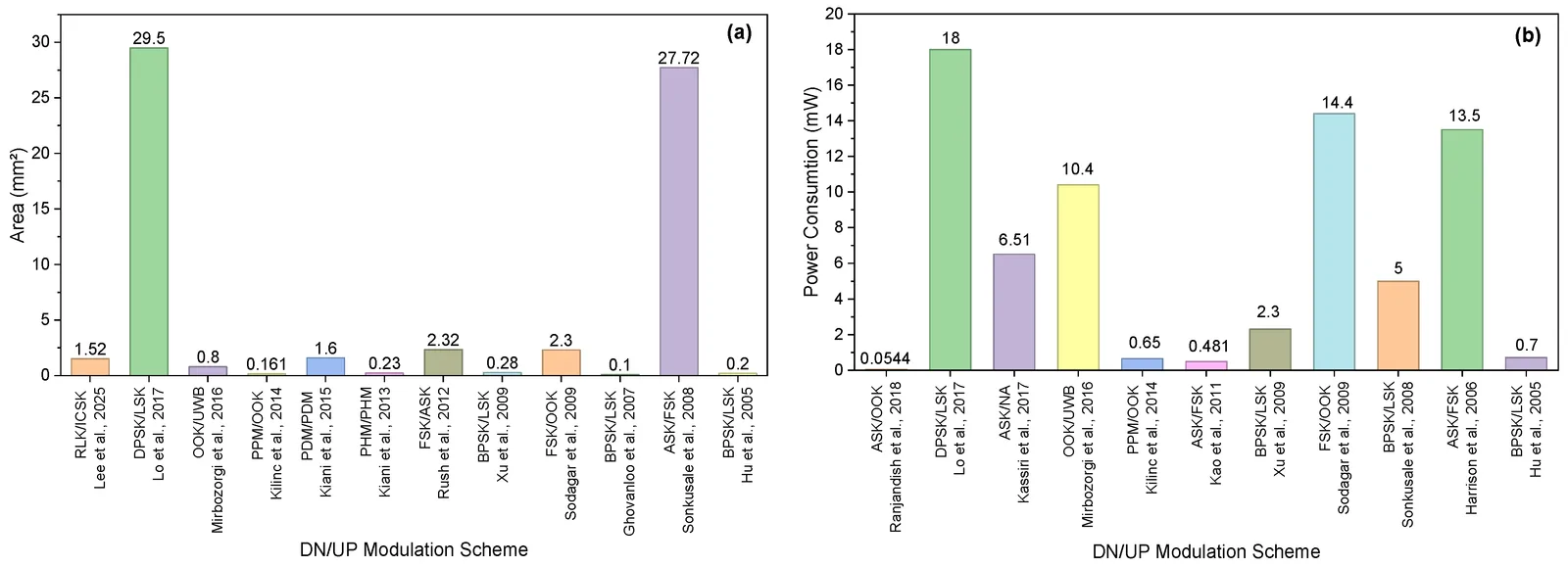
Literature comparison of full-duplex systems reported in [14,32,52,66,67,68,69,71,89,90,91,92,93,94,95,96]: (**a**) area, (**b**) power consumption.

The classification of a full-duplex WPT system is shown in Figure 21 and Figure 22. The highest PTE values for different configurations are reported in [20,66,67]. The highest PDL values with efficient operation are achieved in [14,20,51], respectively. Among them, 61.1% is the highest PTE achieved by the RLK/Instant-CSK-based full-duplex modulation technique [66]. In terms of circuit area, the proposed DPSK/LSK- and ASK/FSK-based circuits have areas of 29.5 ${\;mm}^{2}$ and 27.72 ${mm}^{2}$, the largest sizes among the reported works shown in Figure 22a. BPSK/LSK- [89] and PPM/OOK [90] based prototypes have the smallest sizes of $0.1\;{mm}^{2}$ and $0.161\;{mm}^{2}$.

Figure 22b shows the power consumption of the downlink/uplink data transfer system. The maximum downlink data rate is reported in [14,68,69], while for the uplink, the highest data rate is achieved in [14,69,70]. ASK/OOK [91] and PPM/OOK [90] provide low power consumption due to their simple methods and minimum number of elements utilized.

Detailed experimental results for numerous methods of wireless power and data transfer for full-duplex systems are summarized in Table 3, including carrier frequency, coil distance, PTE, and PDL. The analysis shows that increasing carrier frequency decreases the PTE, and the resonators improve efficiency and allow longer transmission distances.

## 6. Validation Status of Reviewed Systems

Most of the reported WPDT systems using inductive coils are validated through benchtop testing, including [14,20,32,66,68,92,94,95]. Only a few systems have progressed to animal-based in vivo validation, such as flexible RFIC implants validated in rats [18,70], while a neurostimulator for epilepsy monitoring and suppression [67] and a battery-free neuro-electronic interface were tested in a rat model [86]. Moreover, a wireless system-on-chip (SoC) system was validated for motor function recovery in a paralyzed rat [91]. A cat cortex was utilized in [89] for a wireless neural recording system. However, no human clinical validation has been reported in the literature, illustrating an important gap and a key direction for future research.

## 7. Safety Requirements for WPDT Systems

In wireless power and data transfer for implantable medical devices (IMDs), safety constraints are crucial. Among them, biological safety is essential due to direct contact with human tissue. Devices must use biocompatible, chemically stable, and well-encapsulated materials to prevent inflammation, corrosion, and leakage [63,65]. In addition, modulation techniques influence biological safety through passive power dissipation. The choice of modulation technique determines the overall system design, which in turn affects the implant size. Passive modulation schemes allow using a single inductive link to transmit both power and data simultaneously. This makes the circuit less complicated, the device more compact, and the mechanical reliability improved. On the other hand, active telemetry or multi-link setups require additional circuits, which complicate implant structures and could increase the risk of mechanical failure. Electromagnetic safety is primarily concerned with how RF fields from wireless devices damage tissue. Electromagnetic waves pass through biological tissues and are absorbed at different frequencies, potentially causing localized heating. The specific absorption rate (SAR), measured in watts per kilogram (W/kg), quantifies this effect. Two standards are referenced, including IEEE C95.1-1999, which sets the SAR limit for 1 g tissue and states that it should not exceed 1.6 W/kg [98]. Based on the C95.1-2005 standard of IEEE, the SAR for the human body should not be greater than 2 W/kg for 10 g tissue [54]. The SAR limit is 4 W/kg, respectively. For implant power transfer, the reported SAR is 0.4 W/kg for an input power of 0.015 W. The maximum RF exposure level is 10 mW/cm^2^, with power dissipation of 40 mW/cm^2^. According to ISO 14708-1, a general 2 °C maximum temperature rise is specified and serves as the baseline requirement for both cardiac pacemakers and cochlear implants. Moreover, recent studies recommend limiting the tissue temperature increase to 1–2 °C above normal body temperature. It should be noted that most of the studies reviewed in this paper were limited to benchtop or prototype demonstrations; the evaluation of SAR and temperature increase assessment were not in the scope of these studies.

The modulation technique is very important for determining the level of electromagnetic exposure an object experiences. Passive techniques such as PPSK and COOK use the carrier signal generated outside the body to transmit data. This reduces the number of sources within the implant and makes the system more efficient. As in PPSK, the information is encoded in phase rather than amplitude. Active telemetry introduces additional radiated signals, which could increase electromagnetic exposure and make it harder to follow safety rules. Electromagnetic interference (EMI) from components of the circuit itself or from surrounding equipment can degrade the IMD system and put patients at risk. These external electromagnetic fields can induce currents in conductive parts of the implant, causing unwanted heating, or they can actuate magnetic parts, causing mechanical movement or loss of function. The ASK and LSK methods are sensitive to noise, and variations in coupling can easily distort amplitude changes. PPSK is more robust to amplitude variations. COOK makes things even more reliable by maintaining stable resonance behavior and reducing transient distortions during modulation. Active telemetry systems generate increased interference due to the transmission of RF signals at frequencies that overlap with the operating frequency of the system. Additionally, when separate links are utilized for power and data transfer, the EMI effects are made worse.

## 8. Key Challenges & Research Opportunities

Various WPDT techniques aim to transfer power and data between external units and biomedical devices, enabling the devices to operate and communicate with other systems, and vice versa. So, the power transfer needs to be adequate, and the data rate needs to be suitable. This review paper presents several modulation techniques for data encoding. Among them, the CWM and QCWM modulation techniques are said to have a good balance of data rate, size, and power use. However, many challenges need to be addressed in the future to make them suitable for real-world applications. For instance, a fully integrated custom modulator needs to be developed to replace the current COTS-based implementation on the transmitter side to achieve better modulation accuracy and lower power consumption. Choosing a carrier frequency is also crucial for future design, as high carrier frequencies increase sensitivity to parasitic effects and switching losses, which may degrade overall system performance. Low-carrier-frequency-based circuits, such as the 6.78 MHz ISM band, can improve power efficiency and bit error rate (BER) at the cost of reduced data rate. Therefore, achieving an optimal trade-off between efficiency and data rate remains a critical research direction.

Wearable medical devices require a dynamic inductive link capable of supporting a wide range of carrier frequencies. In this framework, integrating a metasurface-based approach into an inductive link is a promising technique to improve power transfer efficiency and enhance coupling under misalignment conditions by controlling the electromagnetic field distribution. However, its effective integration alongside advanced modulation techniques such as CWM and QCWM remains challenging. Furthermore, maintaining continuous power transfer is a crucial concern for modulation schemes such as OOK, CWM, and QCWM due to inherent carrier interruptions. Therefore, the co-design of metasurface and modulation strategies is essential for achieving stable and efficient wireless power and data transfer in next-generation IMDs and wearable medical systems. At the receiver, a demodulator circuit with adaptive voltage-reference control is recommended. It may require microcontroller-assisted calibration to accommodate varying carrier frequencies, as well as the integration of an uplink. Further research is needed into miniaturization via advanced integration to reduce implant and demodulator sizes. Finally, future efforts should investigate hybrid WPT configurations, flexible coil designs, and combined modulation schemes to enhance efficiency, reduce losses, and enable compact, high-data-rate operation for multifunctional biomedical implants.

From a design point of view, passive uplink modulation techniques are clearly better because they use less power, expose users to less electromagnetic radiation, and are easier to install. PPSK and COOK are more robust and efficient than regular LSK, making them better suited for implantable systems that need to operate reliably and safely. A single inductive link with good modulation makes the device smaller, causes less tissue damage, and reduces the likelihood of interference. So, the choice of uplink modulation is not only important for communication design but also for ensuring that biological, physical, and electromagnetic safety standards are met.

## 9. Conclusions

This paper provides a comprehensive review of wireless power and data transfer using inductive coils for IMDs. Recent advancements in IMD miniaturization have led to the development of ultra-small, biocompatible devices that can be implanted with minimally invasive procedures. These devices now feature enhanced battery life and improved functionality, thanks to innovations in low-power consumption technologies. Additionally, the use of advanced materials has further reduced device size while maintaining durability and performance. The WPT systems are systematically categorized into single- and multiple-inductive-link structures based on coil geometries and topologies. Single-inductive-link WPT systems are compact and energy-efficient but offer modest performance. On the other hand, multiple-inductive-link systems offer higher power transfer and data communication capacity, albeit at the cost of greater architectural complexity.

In wireless data transfer, numerous modulation schemes have been reported to transfer power and data to IMDs. A comprehensive review of these techniques has been presented above. Among them, ASK is preferred due to its simple architecture and low power consumption; however, it is highly sensitive to noise. Unlike ASK, FSK provides continuous power transfer at high data rates and is robust to noise. However, due to the complex design structure and the requirement for a wide operating bandwidth, it is rarely utilized. On the other hand, PSK and its derivatives are robust to noise and have higher data rates. However, design complexity and power consumption are the main challenges to their real-world implementation.

This paper focuses on ASK modulation and its derivatives. A fundamental limitation arises from the trade-off between PTE and data rate, governed by the quality factor (Q). A high Q improves PTE but reduces the available bandwidth, thereby limiting the data rate. In addition, traditional ASK demodulators are constrained by the use of envelope detectors. These detectors, typically implemented using a diode and RC network, introduce bandwidth limitations and restrict the achievable data rate. Moreover, the diode’s switching behavior increases power consumption. Based on the same ASK-OOK modulation technique, a novel wideband, comparator-less data-recovery circuit is reported. Unlike the traditional ASK circuit, this design utilizes a PW2SP converter and a comparator-free approach. The measured results of these designs are very encouraging in terms of wideband carrier frequency data rate, power consumption, and compact size.

Additionally, advanced modulation techniques such as CWM and QCWM are proposed to provide high data transmission rates while minimizing power consumption. Their proposed designs utilize a PW2SP converter circuit along with a series of comparators and logic circuits. However, due to the fixed current-mirror circuit in PW2SP, the frequency range is limited. To overcome this issue, our group recently proposed a counter-based QCWM circuit design featuring a programmable current mirror to support a range of carrier frequencies. All of the above works focus on downlink communication.

For uplink data transfer, OOK, LSK, and COOK are recommended as passive telemetry systems because they rely on externally generated carriers and do not require their own power sources, thereby reducing overall power consumption. In LSK, data transfer is achieved by changing the load impedance of the secondary circuit or by capacitive load modulation. Compared to resistive modulation, capacitive switching can induce larger variations in the reflected impedance while dissipating less power. However, this advantage comes at the cost of detuning the LC tank, which leads to off-resonance operation and consequently degrades PTE. In the case of OOK, a maximum data rate of 12 Mb/s has been reported, which is higher than that of other schemes. COOK and PPSK also demonstrate promising data-rate performance, particularly COOK. However, these techniques have received limited attention and offer significant potential for further research, especially in the context of low-power wireless communication and energy transfer systems.

This review of advanced modulation techniques finds that QCWM and COOK provide the highest downlink and uplink communication, respectively, over a single inductive link for both power and data transmission and consume low power. However, for QCWM and COOK, evaluating the BER for different load conditions and distances remains. A single inductive link for both power and data transmission offers many benefits, making the implant smaller and reducing power consumption. But sharing the same inductive link can cause interference that makes PTE worse and makes the circuit transmitter more difficult to use. The selection of the carrier frequency is critical and challenging, balancing key parameters such as data bandwidth, PTE, transmitter and receiver efficiencies, and SAR safety limits.

## Figures and Tables

**Table 2:** Benchmarking of recent uplink data modulation methods.

Ref.	Mod.	Link Architecture	Tech. (µm)	Carrier (MHz)	Data Rate (Mb/s)	Power Consumption (µW)	BER	Max ($P_{DL}$) (mW)	Area (mm^2^)
[20]	PPSK	Single	0.6 (HV)	13.56	1.35	-	<$6\;\times \;10^{-8}$	100	-
[28]	LSK	Single	0.5	25	2.8	100	$10^{-6}$	-	-
[61]	COOK	Single	0.065	13.56	6.78	64	<9.9$\;\times \;10^{-8}$	11.5	0.9
[86]	LSK	Single	Discrete	1	0.0033	-	1$\;\times \;10^{-6}$	250	
[87]	LSK	Single	0.18	10	2	-	-	10	7.74
[88]	LSK	Single	Discrete	13.56	0.5	-	-	20	0.18

**Table 3:** Benchmarking of recent full-duplex data modulation Scheme.

Work	Year	Tech (nm)	Distance	Link Architecture	Frequency (MHz)		Modulation		Data Rate (Mb/s)		Area (mm^2^)	BER (DN/UP)	Efficiency	Power (mW)	
					UP	DN	UP	DN	DN	UP				Deli.	Cons.
[66]	2026	0.18	-	-	-	40.68	Instant-CSK	RLK	3.4	17	1.52	${\leq 10}^{-7}$/${\leq 10}^{-5}$	61.1	-	-
[91]	2018	0.18	10	Single	416	8	00K	ASK	0.5	12	-	-	30	10	0.544
[92]	2017	0.18	-	Single	10	10	LSK	BPSK	1.12	-	29.5	2 × 10^−5^	-	0.61	0.7
[68]	2017	0.13	20	Single	3.1–10.6 G	1.5	-	ASK	-	46	6	1 × 10^−6^	-		6.51
[20]	2016	0.6	8	UWB	13.56	13.56	PPSK	00K	0.4	1.35	-	5.9 × $10^{-8}$	60.64	100	-
[90]	2014	0.18	30	Single	869	13.56	OOK	PPM	0.0066	1.5	0.161	-	16.8	-	0.65
[51]	2014	0.35	10	Single	50	50	PDM	PDM	13.56	13.56	1.6	4.3 × $10^{-7}$	-	42	-
[69]	2013	0.35	10	Single	66.6	66.6	PHM	PHM	20	20	0.23	8.7 × $10^{-8}$	-	-	-
[93]	2012	0.8	20	Single	50–100	5	ASK/BPS	FSK	1.25	3	2.32	-	-		-
[94]	2011	0.18	10	Single	0.2–0.33	2	FSK	ASK	1	0.13 3	-	-	-	-	0.481
[70]	2011	0.5	10	Single	67.5	67.5	PHM	PHM	10.2	10.2	-	6.3 × 10^−6^	-	-	-
[67]	2010	-	-	Single	13.56	13.56	OQPSK	OQPSK	4.16	4.16	-	2 × 10^−6^	61		-
[95]	2008	0.5	20	Single	0.1	13.56	LSK	BPSK	1.69	0.1	0.1			22.5	5
[89]	2007	-	10	-	50	5	PWM	FSK	0.2	-	-	-		-	
[96]	2007	0.5	130	Single	433	2.64	FSK	ASK	0.0065	0.33	27.73	-	13	-	13.5
[97]	2025	0.18	10	Single	-	13.56	SP-LSK	ODSK	0.4	0.4	-	10^−6^/10^−6^	34.8	32	-

## Data Availability

Data sharing is not applicable as no new data were created or analyzed in this paper.

## Abbreviations

The following abbreviations are used in this manuscript:

DN	Downlink
ASK	Amplitude Shift Keying
BER	Bit Error Rate
COOK	Cyclic On-Off Keying
CWM	Carrier Width Modulation
QCWM	Quad-Carrier Width Modulation
FD	Forward Data
FSK	Frequency Shift Keying
IMD	Three-Letter Acronym
LSK	Load Shift Keying
OOK	On-Off Keying
PTE	Power Transfer Efficiency
PSK	Phase Shift Keying
PPSK	Passive Phase-Shift Keying
QAM	Quadrature Amplitude Modulation
SAR	Specific Absorption Rate
SWPDT	Simultaneous Wireless Power and Data Transfer
UP	Uplink
WPT	Wireless Power Transfer

## References

[1] Khan A.N., Cha Y.-O., Giddens H., Hao Y. (2022). Recent Advances in Organ-Specific Wireless Bioelectronic Devices: Perspective on Biotelemetry and Power Transfer Using Antenna Systems. Engineering.

[2] Aboualalaa M., Mansour M., Mansour I. (2025). Wireless Power Transfer for Biomedical Implants. Int. J. Circuit Theory Appl..

[3] Asif S.M., Iftikhar A., Hansen J.W., Khan M.S., Ewert D.L., Braaten B.D. (2019). A Novel RF-Powered Wireless Pacing via a Rectenna-Based Pacemaker and a Wearable Transmit-Antenna Array. IEEE Access.

[4] Yoo S., Lee J., Joo H., Sunwoo S.-H., Kim S., Kim D.-H. (2021). Wireless Power Transfer and Telemetry for Implantable Bioelectronics. Adv. Healthc. Mater..

[5] RamRakhyani A.K., Mirabbasi S., Chiao M. (2011). Design and Optimization of Resonance-Based Efficient Wireless Power Delivery Systems for Biomedical Implants. IEEE Trans. Biomed. Circuits Syst..

[6] Mei H., Thackston K.A., Bercich R.A., Jefferys J.G.R., Irazoqui P.P. (2017). Cavity Resonator Wireless Power Transfer System for Freely Moving Animal Experiments. IEEE Trans. Biomed. Eng..

[7] Abdulfattah A.N., Tsimenidis C.C., Al-Jewad B.Z., Yakovlev A. (2019). Performance Analysis of MICS-Based RF Wireless Power Transfer System for Implantable Medical Devices. IEEE Access.

[8] Lee S.P., Ha G., Wright D.E., Ma Y., Sen-Gupta E., Haubrich N.R., Branche P.C., Li W., Huppert G.L., Johnson M. (2018). Highly flexible, wearable, and disposable cardiac biosensors for remote and ambulatory monitoring. npj Digit. Med..

[9] Koo J., Kim S.B., Choi Y.S., Xie Z., Bandodkar A.J., Khalifeh J., Yan Y., Kim H., Pezhouh M.K., Doty K. (2020). Wirelessly controlled, bioresorbable drug delivery device with active valves that exploit electrochemically triggered crevice corrosion. Sci. Adv..

[10] Raji H., Kumar S., Meng Z., Berthiaume F., Javanmard M. (2025). Wireless Power-Up and Readout of Label-Free Nanosensors for In-Vivo Monitoring of Protein Concentrations in Live Animals. Langmuir.

[11] Mondal S., Zehra N., Choudhury A., Iyer P.K. (2020). Wearable Sensing Devices for Point of Care Diagnostics. ACS Appl. Bio Mater..

[12] Wang J., Bai J., Ma C.-B., Gu M., Sun M., Zhou M. (2026). Wearable Nanosensor for Noninvasive Multimodal Monitoring of Theophylline Pharmacokinetics and Hemodynamics. ACS Sens..

[13] Liu W., Sivaprakasam M., Wang G., Zhou M., Granacki J., Lacoss J., Wills J. (2005). Implantable biomimetic microelectronic systems design. IEEE Eng. Med. Biol. Mag..

[14] Mirbozorgi S.A., Bahrami H., Sawan M., Rusch L.A., Gosselin B. (2016). A Single-Chip Full-Duplex High Speed Transceiver for Multi-Site Stimulating and Recording Neural Implants. IEEE Trans. Biomed. Circuits Syst..

[15] Gong C., Liu D., Miao Z., Li M. (2017). A Magnetic-Balanced Inductive Link for the Simultaneous Uplink Data and Power Telemetry. Sensors.

[16] Atluri S., Ghovanloo M. Design of a Wideband Power-Efficient Inductive Wireless Link for Implantable Biomedical Devices Using Multiple Carriers. Proceedings of the 2nd International IEEE EMBS Conference on Neural Engineering.

[17] Kaim V., Singh N., Kanaujia B.K., Matekovits L., Esselle K.P., Rambabu K. (2023). Multi-Channel Implantable Cubic Rectenna MIMO System with CP Diversity in Orthogonal Space for Enhanced Wireless Power Transfer in Biotelemetry. IEEE Trans. Antennas Propag..

[18] Hwang G.-T., Im D., Lee S.E., Lee J., Koo M., Park S.Y., Kim S., Yang K., Kim S.J., Lee K. (2013). In Vivo Silicon-Based Flexible Radio Frequency Integrated Circuits Monolithically Encapsulated with Biocompatible Liquid Crystal Polymers. ACS Nano.

[19] Uehlin J.P., Smith W.A., Pamula V.R., Pepin E.P., Perlmutter S., Sathe V., Rudell J.C. (2020). A Single-Chip Bidirectional Neural Interface with High-Voltage Stimulation and Adaptive Artifact Cancellation in Standard CMOS. IEEE J. Solid-State Circuits.

[20] Jiang D., Cirmirakis D., Schormans M., Perkins T.A., Donaldson N., Demosthenous A. (2017). An Integrated Passive Phase-Shift Keying Modulator for Biomedical Implants with Power Telemetry over a Single Inductive Link. IEEE Trans. Biomed. Circuits Syst..

[21] Li X., Tsui C.-Y., Ki W.-H. (2015). A 13.56 MHz Wireless Power Transfer System with Reconfigurable Resonant Regulating Rectifier and Wireless Power Control for Implantable Medical Devices. IEEE J. Solid-State Circuits.

[22] Kiani M., Ghovanloo M. (2010). An RFID-Based Closed-Loop Wireless Power Transmission System for Biomedical Applications. IEEE Trans. Circuits Syst. II Express Briefs.

[23] Trigui A., Hached S., Ammari A.C., Savaria Y., Sawan M. (2019). Maximizing Data Transmission Rate for Implantable Devices over a Single Inductive Link: Methodological Review. IEEE Rev. Biomed. Eng..

[24] Karimi M.J., Schmid A., Dehollain C. (2021). Wireless Power and Data Transmission for Implanted Devices via Inductive Links: A Systematic Review. IEEE Sens. J..

[25] Hong B., Wei X., Shi C., Mai S., Tang X., Wang Z. (2026). A review of wireless power and data transfer systems for biomedical implants. Neuroelectronics.

[26] Baker M.W., Sarpeshkar R. (2007). Feedback Analysis and Design of RF Power Links for Low-Power Bionic Systems. IEEE Trans. Biomed. Circuits Syst..

[27] Sample A.P., Meyer D.A., Smith J.R. (2011). Analysis, Experimental Results, and Range Adaptation of Magnetically Coupled Resonators for Wireless Power Transfer. IEEE Trans. Ind. Electron..

[28] Yilmaz G., Dehollain C. (2014). Single frequency wireless power transfer and full-duplex communication system for intracranial epilepsy monitoring. Microelectron. J..

[29] Garcerán-Hernández V., Martínez-Rams E.A. (2013). Cochlear Implant: Transcutaneous Transmission Link with OFDM. Natural and Artificial Models in Computation and Biology.

[30] Sawan M., Hu Y., Coulombe J. (2005). Wireless smart implants dedicated to multichannel monitoring and microstimulation. IEEE Circuits Syst. Mag..

[31] Ye D., Wang Y., Xiang Y., Lyu L., Min H., Shi C.-J.R. (2020). A Wireless Power and Data Transfer Receiver Achieving 75.4% Effective Power Conversion Efficiency and Supporting 0.1% Modulation Depth for ASK Demodulation. IEEE J. Solid-State Circuits.

[32] Hu Y., Sawan M. (2005). A fully integrated low-power BPSK demodulator for implantable medical devices. IEEE Trans. Circuits Syst. I Regul. Pap..

[33] Ghovanloo M., Najafi K. A high-rate frequency shift keying demodulator chip for wireless biomedical implants. Proceedings of the 2003 International Symposium on Circuits and Systems, 2003. ISCAS ’03.

[34] Knecht O., Kolar J.W. (2019). Performance Evaluation of Series-Compensated IPT Systems for Transcutaneous Energy Transfer. IEEE Trans. Power Electron..

[35] Elsayed E.E. (2024). Investigations on modified OOK and adaptive threshold for wavelength division multiplexing free-space optical systems impaired by interchannel crosstalk, atmospheric turbulence, and ASE noise. J. Opt..

[36] Trigui A., Ali M., Ammari A.C., Savaria Y., Sawan M. (2019). Energy Efficient Generic Demodulator for High Data Transmission Rate over an Inductive Link for Implantable Devices. IEEE Access.

[37] Liao Z.-J., Sun Y., Ye Z.-H., Tang C.-S., Wang P.-Y. (2019). Resonant Analysis of Magnetic Coupling Wireless Power Transfer Systems. IEEE Trans. Power Electron..

[38] Aditya K., Williamson S.S. (2019). Design Guidelines to Avoid Bifurcation in a Series–Series Compensated Inductive Power Transfer System. IEEE Trans. Ind. Electron..

[39] Ahn D., Hong S. (2013). Effect of Coupling Between Multiple Transmitters or Multiple Receivers on Wireless Power Transfer. IEEE Trans. Ind. Electron..

[40] Lee W.-S., Son W.-I., Oh K.-S., Yu J.-W. (2013). Contactless Energy Transfer Systems Using Antiparallel Resonant Loops. IEEE Trans. Ind. Electron..

[41] Lyu Y.-L., Meng F.-Y., Yang G.-H., Che B.-J., Wu Q., Sun L., Erni D., Li J.L.-W. (2015). A Method of Using Nonidentical Resonant Coils for Frequency Splitting Elimination in Wireless Power Transfer. IEEE Trans. Power Electron..

[42] Lee J., Lim Y.-S., Yang W.-J., Lim S.-O. (2014). Wireless Power Transfer System Adaptive to Change in Coil Separation. IEEE Trans. Antennas Propag..

[43] Kim J., Jeong J. (2015). Range-Adaptive Wireless Power Transfer Using Multiloop and Tunable Matching Techniques. IEEE Trans. Ind. Electron..

[44] Dang Z., Abu Qahouq J.A. Extended-range two-coil adaptively reconfigurable wireless power transfer system. Proceedings of the 2015 IEEE Applied Power Electronics Conference and Exposition (APEC).

[45] Jeong S., Lin T.-H., Tentzeris M.M. (2019). A Real-Time Range-Adaptive Impedance Matching Utilizing a Machine Learning Strategy Based on Neural Networks for Wireless Power Transfer Systems. IEEE Trans. Microw. Theory Tech..

[46] Mai R., Yue P., Liu Y., Zhang Y., He Z. (2018). A Dynamic Tuning Method Utilizing Inductor Paralleled with Load for Inductive Power Transfer. IEEE Trans. Power Electron..

[47] Ullah N., Barakat A., Kanaya H., Pokharel R.K. (2025). Wideband ASK-OOK Data Recovery Circuit for Data Transmission in Over-Coupled Mode of SWPDT System. Electronics.

[48] Ullah N., Zulkalnain M., Barakat A., Kanaya H., Pokharel R.K. (2025). A Comparator-Less ASK-OOK Data Demodulator Circuit for Downlink Data Transfer over Inductive Link. Int. J. Circuit Theory Appl..

[49] Bazaka K., Jacob M. (2012). Implantable Devices: Issues and Challenges. Electronics.

[50] Mandal S., Sarpeshkar R. (2008). Power-Efficient Impedance-Modulation Wireless Data Links for Biomedical Implants. IEEE Trans. Biomed. Circuits Syst..

[51] Kiani M., Ghovanloo M. (2015). A 13.56-Mbps Pulse Delay Modulation Based Transceiver for Simultaneous Near-Field Data and Power Transmission. IEEE Trans. Biomed. Circuits Syst..

[52] Liang C.-K., Chen J.-J.J., Chung C.-L., Cheng C.-L., Wang C.-C. (2005). An implantable bi-directional wireless transmission system for transcutaneous biological signal recording. Physiol. Meas..

[53] Puers R., Thoné J. (2010). Short Distance Wireless Communications. Bio-Medical CMOS ICs.

[54] Trigui A., Hached S., Mounaim F., Ammari A.C., Sawan M. (2015). Inductive Power Transfer System with Self-Calibrated Primary Resonant Frequency. IEEE Trans. Power Electron..

[55] Troyk P.R., Cogan S.F. (2005). Sensory Neural Prostheses. Neural Engineering.

[56] Trigui A., Ali M., Ammari A.C., Savaria Y., Sawan M. (2018). A 1.5-pJ/bit, 9.04-Mbit/s Carrier-Width Demodulator for Data Transmission over an Inductive Link Supporting Power and Data Transfer. IEEE Trans. Circuits Syst. II Express Briefs.

[57] Trigui A., Ali M., Ammari A.C., Savaria Y., Sawan M. Quad-Level Carrier Width Modulation demodulator for micro-implants. Proceedings of the 2016 14th IEEE International New Circuits and Systems Conference (NEWCAS).

[58] Zulkalnain M.K., Barakat A., Ullah N., Kanaya H., Pokharel R.K. (2025). Counter-based CMOS QCWM demodulator for wide frequency range WPT biohealth applications. Mem.-Mater. Devices Circuits Syst..

[59] Khanna V.K. (2016). Wireless Communications and Powering of Implants. Implantable Medical Electronics.

[60] Bawa G., Ghovanloo M. (2008). Active High Power Conversion Efficiency Rectifier with Built-In Dual-Mode Back Telemetry in Standard CMOS Technology. IEEE Trans. Biomed. Circuits Syst..

[61] Ha S., Kim C., Park J., Joshi S., Cauwenberghs G. (2016). Energy Recycling Telemetry IC with Simultaneous 11.5 mW Power and 6.78 Mb/s Backward Data Delivery over a Single 13.56 MHz Inductive Link. IEEE J. Solid-State Circuits.

[62] Gong C., Liu D., Miao Z., Wang W., Li M. (2017). An NFC on Two-Coil WPT Link for Implantable Biomedical Sensors under Ultra-Weak Coupling. Sensors.

[63] Hu Y., Gervais J.-F., Sawan M. High power efficiency inductive link with full-duplex data communication. Proceedings of the 9th International Conference on Electronics, Circuits and Systems.

[64] Xu W., Luo Z., Sonkusale S. (2009). Fully Digital BPSK Demodulator and Multilevel LSK Back Telemetry for Biomedical Implant Transceivers. IEEE Trans. Circuits Syst. II Express Briefs.

[65] Catrysse M., Hermans B., Puers R. (2004). An inductive power system with integrated bi-directional data-transmission. Sens. Actuators A Phys..

[66] Lee J., Kim Y., Kang D., Song I., Lee B. (2025). A Reconfigurable Bidirectional Wireless Power and Full-Duplex Data Transceiver IC for Wearable Biomedical Applications. IEEE Trans. Biomed. Circuits Syst..

[67] Simard G., Sawan M., Massicotte D. (2010). High-Speed OQPSK and Efficient Power Transfer Through Inductive Link for Biomedical Implants. IEEE Trans. Biomed. Circuits Syst..

[68] Kassiri H., Salam M.T., Pazhouhandeh M.R., Soltani N., Velazquez J.L.P., Carlen P., Genov R. (2017). Rail-to-Rail-Input Dual-Radio 64-Channel Closed-Loop Neurostimulator. IEEE J. Solid-State Circuits.

[69] Kiani M., Ghovanloo M. (2013). A 20-Mb/s Pulse Harmonic Modulation Transceiver for Wideband Near-Field Data Transmission. IEEE Trans. Circuits Syst. II Express Briefs.

[70] Inanlou F., Kiani M., Ghovanloo M. (2011). A 10.2 Mbps Pulse Harmonic Modulation Based Transceiver for Implantable Medical Devices. IEEE J. Solid-State Circuits.

[71] Sodagar A.M., Perlin G.E., Yao Y., Najafi K., Wise K.D. (2009). An Implantable 64-Channel Wireless Microsystem for Single-Unit Neural Recording. IEEE J. Solid-State Circuits.

[72] Ture K., Kilinc E.G., Maloberti F., Dehollain C. (2016). Remotely powered PPM demodulator by inductive coupling for rodent applications. Analog. Integr. Circuits Signal Process..

[73] Troyk P.R., Edgington M. Inductive links and drivers for remotely-powered telemetry systems. Proceedings of the IEEE Antennas and Propagation Society International Symposium. Transmitting Waves of Progress to the Next Millennium. 2000 Digest. Held in Conjunction with: USNC/URSI National Radio Science Meeting (Cat. No.00CH37118).

[74] Gong C.-S.A., Yao K.-W., Shiue M.-T., Lin P.-Y., Huang C.-N. A Miniaturized PSK Demodulation Device for Short-Range Wireless Neural Prosthetic Systems. Proceedings of the 2008 2nd International Conference on Bioinformatics and Biomedical Engineering.

[75] Lee H.-S., Lee H.-M. (2025). A Power-Efficient Envelope-Detector-Less Amplitude-Shift-Keying Forward Telemetry for Wirelessly Powered Biomedical Devices. IEEE Trans. Biomed. Circuits Syst..

[76] Park Y., Hung P.D., Youn D., Kwon D., Kim C., Je M. An Enhanced-Frequency-Splitting-Based Wireless Power and Data Transfer System Achieving 60.2% End-to-End Efficiency and 1 Mb/s Data Rate with a Sub-cm RX Coil for Miniaturized Implants. Proceedings of the 2025 IEEE International Solid-State Circuits Conference (ISSCC).

[77] Chen Y., Liu Y., Li Y., Wang G., Chen M. (2022). An Energy-Efficient ASK Demodulator Robust to Power-Carrier-Interference for Inductive Power and Data Telemetry. IEEE Trans. Biomed. Circuits Syst..

[78] Lee H., Kim J., Ha D., Kim T., Kim S. (2015). Differentiating ASK Demodulator for Contactless Smart Cards Supporting VHBR. IEEE Trans. Circuits Syst. II Express Briefs.

[79] Mazzilli F., Dehollain C. (2016). 184 μW ultrasonic on–off keying/amplitude-shift keying demodulator for downlink communication in deep implanted medical devices. Electron. Lett..

[80] Navidi M.M., Byun G.-S. A near-threshold ASK demodulator for ultra-low-power implantable biomedical microsystems. Proceedings of the WAMICON 2013.

[81] Park Y., Dang Hung P., Youn D., Kwon D., Kim C., Je M. (2025). A Wireless Power and Data Transfer System for Medical Implants Using a Miniaturized Inductive Link with Frequency-Splitting Enhancement. IEEE J. Solid-State Circuits.

[82] Qian C., Li S., Liang H., Zhang H., Mai S. (2026). A Dual-mode FSK Wireless Power and Data Transfer System with 1.1 Mbps Data Rate and Rectifier Output Power of 148 mW for Implantable Medical Applications. IEEE Trans. Biomed. Circuits Syst..

[83] Ullah N., Barakat A., Zulkalnain M., Kanaya H., Pokharel R.K. (2025). Compact and Efficient FSK-CDR Design for Forward Data Transfer over Wireless Inductive Link. Int. J. Circuit Theory Appl..

[84] Qu Y., Zhang B., Gu W., Li J., Shu X. (2023). Distance Extension of S-PS Wireless Power Transfer System Based on Parity-Time Symmetry. IEEE Trans. Circuits Syst. II Express Briefs.

[85] Park Y., Koh S.-T., Lee J., Kim H., Choi J., Ha S., Kim C., Je M. 33.7 A Frequency-Splitting-Based Wireless Power and Data Transfer IC for Neural Prostheses with Simultaneous 115mW Power and 2.5Mb/s Forward Data Delivery. Proceedings of the 2021 IEEE International Solid-State Circuits Conference (ISSCC).

[86] Wang G., Liu W., Sivaprakasam M., Kendir G.A. (2005). Design and analysis of an adaptive transcutaneous power telemetry for biomedical implants. IEEE Trans. Circuits Syst. I Regul. Pap..

[87] Lin Y.-P., Yeh C.-Y., Huang P.-Y., Wang Z.-Y., Cheng H.-H., Li Y.-T., Chuang C.-F., Huang P.-C., Tang K.-T., Ma H.-P. (2016). A Battery-Less, Implantable Neuro-Electronic Interface for Studying the Mechanisms of Deep Brain Stimulation in Rat Models. IEEE Trans. Biomed. Circuits Syst..

[88] Lee H.-M., Ghovanloo M. (2011). An Integrated Power-Efficient Active Rectifier with Offset-Controlled High Speed Comparators for Inductively Powered Applications. IEEE Trans. Circuits Syst. I Regul. Pap..

[89] Ghovanloo M., Atluri S. (2007). A Wide-Band Power-Efficient Inductive Wireless Link for Implantable Microelectronic Devices Using Multiple Carriers. IEEE Trans. Circuits Syst. I Regul. Pap..

[90] Kilinc E.G., Dehollain C., Maloberti F. A low-power PPM demodulator for remotely powered batteryless implantable devices. Proceedings of the 2014 IEEE 57th International Midwest Symposium on Circuits and Systems (MWSCAS).

[91] Ranjandish R., Ture K., Maloberti F., Dehollain C., Schmid A. All Wireless, 16-Channel Epilepsy Control System with Sub-µW/Channel and Closed-Loop Stimulation Using a Switched-Capacitor-Based Active Charge Balancing Method. Proceedings of the ESSCIRC 2018—IEEE 44th European Solid State Circuits Conference (ESSCIRC).

[92] Lo Y.-K., Kuan Y.-C., Culaclii S., Kim B., Wang P.-M., Chang C.-W., Massachi J.A., Zhu M., Chen K., Gad P. (2017). A Fully Integrated Wireless SoC for Motor Function Recovery After Spinal Cord Injury. IEEE Trans. Biomed. Circuits Syst..

[93] Rush A.D., Troyk P.R. (2012). A Power and Data Link for a Wireless-Implanted Neural Recording System. IEEE Trans. Biomed. Eng..

[94] Kao C.-H., Lin Y.-P., Tang K.-T. Wireless data and power transmission circuits in biomedical implantable applications. Proceedings of the International Symposium on Bioelectronics and Bioinformations.

[95] Sonkusale S., Luo Z. A complete data and power telemetry system utilizing BPSK and LSK signaling for biomedical implants. Proceedings of the 2008 30th Annual International Conference of the IEEE Engineering in Medicine and Biology Society.

[96] Harrison R.R., Watkins P.T., Kier R.J., Lovejoy R.O., Black D.J., Greger B., Solzbacher F. (2007). A Low-Power Integrated Circuit for a Wireless 100-Electrode Neural Recording System. IEEE J. Solid-State Circuits.

[97] Lee J., Kim Y., Kim D., Kang D., Song M., Lee B. A Wireless Power and Synchronized Full-Duplex Data Transceiver IC with 400 Kbps Bidirectional Data Rate Using a Single Inductive Link for Low-Power Systems. Proceedings of the 2025 Symposium on VLSI Technology and Circuits (VLSI Technology and Circuits).

[98] (1999). IEEE Standard for Safety Levels with Respect to Human Exposure to Radio Frequency Electromagnetic Fields, 3 kHz to 300 GHz.

